# A longitudinal investigation of the acoustic properties of infant-directed speech from 6 to 18 months

**DOI:** 10.1098/rsos.240572

**Published:** 2024-11-08

**Authors:** Audun Rosslund, Julien Mayor, Roger Mundry, Arun Prakash Singh, Alejandrina Cristia, Natalia Kartushina

**Affiliations:** ^1^ Center for Multilingualism in Society across the Lifespan, University of Oslo, Oslo, Norway; ^2^ Department of Psychology, University of Oslo, Oslo, Norway; ^3^ Cognitive Ethology Laboratory, German Primate Center, Leibniz Institute for Primate Research, Göttingen, Germany; ^4^ Department for Primate Cognition, Johann-Friedrich-Blumenbach Institute, Georg-August-University Göttingen, Göttingen, Germany; ^5^ Leibniz ScienceCampus Primate Cognition, Göttingen, Germany; ^6^ Department of Cognitive Studies, Paris Sciences et Lettres University, Paris, France

**Keywords:** infant-directed speech, acoustic analysis, language acquisition, longitudinal

## Abstract

Caregivers often modulate their speech when interacting with infants, adapting a register that has been suggested to have attentional, affective and didactic purposes. The present preregistered study examined the longitudinal trajectories of a diverse range of acoustic features of infant-directed speech (IDS) and compared these with adult-directed speech (ADS), in Norwegian parents of 6- to 18-month-old infants. Sixty-nine families participated. Throughout five laboratory visits across one year, parents were recorded reading a picture-book to their infant (IDS) and an experimenter (ADS). The book was designed to tightly control for the linguistic content and context of speech between participants, timepoints and registers. Analyses of a total of 54 594 vowels and 22 958 phrases revealed, first, an overall effect of register: parents used higher pitch, wider pitch range, slower articulation rate, longer vowel duration and more variable and less distinct vowels in IDS than in ADS. Second, significant register-by-age interactions indicated that parents’ IDS, compared with their ADS, featured wider pitch range, larger vowel space and shorter vowel duration in older as compared with younger infants, while pitch, articulation rate and vowel variability and distinctiveness remained relatively stable with age. Results are discussed in the context of the proposed functions of IDS.

## 1. Introduction

Parents tend to adjust the acoustic properties of speech when addressing their infants. Compared with adult-directed speech (ADS), infant-directed speech (IDS) is a specialized speech register typically expressed with higher and more variable pitch, slower articulation rate and longer vowels with more peripheral articulation that expands the vowel space area [1,2]; meta-analysis in [3]. Certain aspects of this prototypical acoustic profile are found in IDS across most languages and cultures studied to date [4,5] (but see [6]),^1^ and IDS is readily distinguished from ADS by naive adult listeners [10]. Although that much is clear, less is known with respect to whether, and if so how, parents’ IDS changes across their child’s development, since longitudinal studies on the acoustic properties of IDS are sparse, and, so far, their results are conflicting. In the current study, we examine such changes across a diverse range of acoustic measures, in a longitudinal sample of Norwegian parent–infant dyads.

The acoustic modifications of IDS are primarily considered to have three functions: (i) to attract and sustain infants’ attention [11–16], (ii) to convey and regulate affect and foster socio-emotional bonding [17–23], and (iii) to support infants’ linguistic development [24–27] (but see [28]). While these functions are not mutually exclusive,^2^ it has been suggested that their relative importance shifts with infants’ age: IDS as a tool to capture attention and convey emotional valence might be the main function in speech directed to young infants, while its facilitating role in language learning might emerge later in development [30–33].

In line with this view, studies have demonstrated that infants’ preference for specific properties of IDS varies with age. For example, Panneton *et al*. [34] found that Australian English 4-month-old infants preferred slower speech and speech that was high in positive vocal affect (as judged by adult listeners), while 8-month-old infants preferred speech with normal tempo, regardless of the vocal affect. Kitamura & Notley [35] reported that Australian English 6-month-olds, but not 10-month-olds, listened longer to words with increased vowel duration and also to words with more exaggerated pitch contours (here, bell-shaped versus monotonic). This could indicate a shift in infants’ attention towards certain properties of speech; that is, moving away from prosody, which is instrumental in conveying emotion [36,37] and sustaining attention and towards features that are more linguistically relevant and could be beneficial to speech processing [38–40] (but see [41]).

Analogously, in a meta-analysis by Spinelli *et al*. [33], it was found that prosodic properties of IDS were more strongly associated with infants’ attentional (*r* = 0.20) and pre-linguistic (*r* = 0.39) outcomes (such as imitation and vocal responsiveness), as compared with linguistic (*r* = 0.17) outcomes (such as word recognition and vocabulary size). Note that this meta-analysis primarily included studies with infants younger than 10 months. While there are studies that report facilitating effects of IDS prosody on linguistic skills in older infants (e.g. [42,43]), these effects could be confounded with other segmental features present in the stimuli, such as vowel space expansion, which has been associated with older infants’ vocabulary size [44] and word recognition [32]. In fact, a recent experimental study reported that Australian English 18-month-old infants showed better word recognition accuracy when exposed to stimuli containing vowel space expansion without prosodic exaggeration as compared with stimuli containing prosodic exaggeration and without vowel space expansion [45].

In sum, these studies suggest a differentiating role of infants’ age on their attentional allocation to specific acoustic properties of IDS and on the association between specific acoustic properties of IDS and infants’ developmental outcomes. Hence the question arises whether parents adapt and fine-tune their IDS in synchronization with infants’ maturing social, cognitive and linguistic competencies and developmental needs. A first step towards answering this question is to examine, longitudinally, how properties of IDS unfold across development. In what follows, we briefly outline the current longitudinal evidence for age-related changes in intonational (pitch, pitch range), temporal (articulation rate and vowel duration) and segmental (vowel space and vowel variability/distinctiveness) components of IDS compared with ADS, addressed to typically developing infants (see table 1 for a definition of these acoustic variables). What will become evident is that results for most acoustic features are not uniform.

**Table 1.** Overview of acoustic measures examined in the current study and their predicted between-register differences and age-related trajectories.

	description	overall difference	trajectory of IDS
pitch	the tonal height of the voice, determined by vocal fold vibrations, measured as the mean fundamental frequency (in Hz) across a phrase, and converted to semitones to align with human auditory perception of speech	higher in IDS	decrease
pitch range	the tonal range of the voice, measured as the difference between the highest and lowest pitch values (in semitones) in a phrase	wider in IDS	stable
articulation rate	the tempo of speech, measured as the number of syllables in a phrase controlled for its phonation time, i.e. the duration of the phrase after excluding any silences	slower in IDS	increase
vowel duration	the length of vowels, measured as the duration of a vowel in milliseconds	longer in IDS	decrease
vowel space area	the articulatory space vowels are produced in, measured as the size of the underlying two-dimensional triangular or polygon area (in Hz^2^) of the mean first and second formant values between point vowels or all border vowels	increased in IDS	stable
vowel variability	the precision in the production of each vowel category, measured as the size of the underlying elliptical area (in Hz^2^) of the first and second formants for each vowel category based on all its tokens	increased in IDS	decrease
vowel distinctiveness	the separateness of vowel categories from each other, measured as the proportion of variance (in quotients) of the first and second formant values that is explained by vowel category membership	decreased in IDS	increase

Higher pitch and wider pitch range are two of the features that are most consistently reported in IDS [3,24,46]. Longitudinal evidence for changes in pitch and pitch range with infants’ age is mixed, with some studies reporting a decrease in the difference between IDS and ADS across development for both intonational measures (Japanese: [47]; Dutch, Mandarin: [48]; American English: [49–52]; German: [53]) and others stability in IDS–ADS difference (Dutch: [17]; Australian English: [54]; American English: [50]; Tamil, Tagalog, Korean: [55]; German: [53]). Synthesizing on longitudinal as well as cross-sectional studies, a recent meta-analysis by Cox *et al*. [3] indicates support for decrease in pitch, but stability in pitch range, across the first 3 years of life.

Temporal features of speech affect the ease of processing for the receiver: fast articulation makes sound segmentation and overall comprehension challenging, whereas elongated vowels increase their identification accuracy [56–58]. Longitudinal studies to date uniformly suggest that articulation rate in IDS increases with infants’ age, becoming more similar to ADS (American English: [49,59]; Australian English: [60]; Tamil, Tagalog, Korean: [55]). Evidence is less consistent with respect to changes in vowel duration, with reports of decrease (Norwegian: [61]; Australian English: [44]; American English: [52]), but also stability (American English: [50]) in differences between IDS and ADS over development. In their meta-analysis, Cox *et al*. [3] found evidence for an increase in articulation rate (infants’ age range in studies = 0−28 months) and a decrease in vowel duration (infants’ age range in studies = 0−24 months).

Finally, turning to segmental features, expansion of the vowel space, traditionally used as an acoustic proxy for ‘hyperarticulation’ (but note that hyperarticulation and vowel space expansion are often used interchangeably in IDS research), is considered to render speech clearer [56] (but see [62]). Exaggeration of the most peripheral vowels (such as /i/-/a/-/u/) expands the underlying vowel space and increases the distance between vowels [2,63]. Age-related changes in relative vowel space area in IDS were not found consistently, with studies showing increases (British English, Japanese, French: [64]), decreases (Australian English: [65]), and stability (Dutch: [17]; Australian English: [54,66]; German: [53]) across infants’ ages—the latter is also the conclusion of two separate meta-analyses: Cox *et al.* [3] (infants’ age range in studies = 0−25 months) and Lovcevic *et al.* [67] (infants’ age range in studies = 0−24 months).

While expansion of the vowel space is considered to make the speech signal clearer by maximizing the distance between vowel categories, there is also evidence that, in IDS, vowels have greater within-category variability and are overall less distinct, as compared with ADS [68–73]. In other words, perhaps resulting in less precise speech, challenging the ‘clarity’ view of IDS brought on by considering vowel space expansion alone. Few longitudinal studies have examined vowel variability and distinctiveness of vowel categories, but the emerging trend appears to be a decrease of variability and increase in distinctiveness with infants’ age. For example, Ratner [74] found that vowel variability, measured as the s.d. of F1 and F2 formants for individual vowels, decreased in the IDS of American English parents as infants developed from the pre-verbal to one-word stage and were less variable in IDS (than ADS) to infants who produced multiple word utterances.^3^ Hartman *et al*. [44] examined vowel variability in American English parents’ IDS when their infants were 11, 18 and 24 months and reported no significant effect of age, yet, vowel variability decreased numerically from 11 to 24 months, becoming more similar to ADS. Extending our review to cross-sectional evidence, Rosslund and colleagues, using the same design and analytical approach, found, in two separate studies, less within-category variability and more distinctive categories in IDS versus ADS directed to Norwegian 18-month-olds (2022), as compared to 8-month-olds (2023). However, a recent study with Danish-learning 11- to 24-month-olds reported no effect of age on vowel distinctiveness between IDS and ADS [68].

In sum, longitudinal studies to date do not paint a clear picture of age-related changes in intonational, temporal and segmental acoustic properties of IDS across infancy. One notable exception is articulation rate, which was consistently reported to increase with age and approach ADS across all reviewed studies (but see also [75]). While conflicting results could be related to differences in cultural and linguistic characteristics of the study samples (note that the majority comprise American- or Australian-English-speaking participants), a set of methodological differences and limitations also suggests caution before interpreting the overall lack of between-study uniformity. For one, studies often use different tasks to elicit IDS and ADS, with little or no systematic control of the linguistic contexts across registers, speakers and timepoints. This makes it challenging to isolate the impact of differences in register from other potential factors influencing speech production, such as consonant context, lexical (word) context and within-sentence position (e.g. [50,76–79]). Additionally, the use of a limited set of objects to elicit IDS in semi-structured tasks does not only hinder extrapolation of results to broader linguistic contexts—it might also more easily induce specific linguistic behaviours in participants [80]. For example, given only three objects, parents may overemphasize contrastive cues when labelling these to their infant, biasing a measure such as vowel space expansion. Crucially, for several longitudinal studies, sampling between registers is asymmetrical; IDS tends to be more frequently recorded, and sometimes at different timepoints, than the ADS that is used for its comparison, that is typically collected only once. The acoustic properties of speech can change due to a range of factors, such as sleep deprivation [81], mental and physical fatigue [82–84], menstrual cycle [85] and post-pregnancy hormonal changes [86] (but see [87]), potentially skewing comparisons between IDS and ADS if registers are sampled at different timepoints. Further, most studies report a limited set of acoustic measures (failing to reveal potential within-parent interactions between changes in measures); often a limited time interval (capturing only brief moments in infant development); and notably, with a limited sample size. The number of participants in the longitudinal studies mentioned above ranged from 3 to 42, with a mean of 17 and a median of 15. While the density of the data within each participant in these studies can still be high, the generalizability of findings remains limited. Considering these limitations that might affect the generalizability of study results, it might be too premature to suggest an association (or a lack thereof) between infants’ preferences and developmental needs of specific components of the speech input provided to them and their parents’ acoustic adaptations in IDS.

The current study addresses the above-mentioned limitations and examines age-related changes in the acoustic properties of IDS and ADS in a cohort of Norwegian parents from 69 different families across one year of their infants’ development, from 6 to 18 months of age, with a 3-month interval between each assessment. Parents’ speech was elicited from a picture-book reading task, to control the quantity and the quality of the linguistic context across registers and speakers and provide a wide variety of target vowels in different within-sentence positions. We assessed a battery of traditionally reported acoustic measures of IDS, namely pitch, pitch range, articulation rate, vowel duration and vowel space area (e.g. [2,46]), but also novel measures of vowel variability and vowel distinctiveness, first reported in Rosslund *et al*. [73]. To the best of our knowledge, this is the first longitudinal study on IDS that (i) examines such a wide range of acoustic measures, (ii) does so in a consistent context across registers and timepoints, (iii) collects ADS that is sampled as frequently, and on the same date, as IDS, (iv) employs a larger sample size compared with previous studies, (v) adopts preregistered hypotheses and analytical pipelines [88,89], and (vi) uses a full-null model comparison approach to minimize type-I errors that can arise from multiple testing [90].

In line with previous above-reviewed research and meta-analytic evidence (where available), and per our detailed preregistration (https://osf.io/mbuzs), we expected that parents’ IDS, compared with ADS, would be characterized by overall higher pitch, wider pitch range, slower articulation rate, longer vowel duration, increased vowel spaces and more variable and less distinct vowel categories [3,31,67,71–73]. Crucially, we expected that the predicted IDS–ADS differences in pitch, articulation rate, vowel duration, vowel category variability and vowel distinctiveness would decrease with infants’ age, attributed to IDS becoming more similar to ADS [3,31,73,74]. In contrast, we expected that the IDS–ADS differences in pitch range and vowel space areas would remain stable with infants’ age [3]. Note that we did not predict changes in ADS with infants’ age. Finally, we expected that the above age-related trajectories of IDS would be more prominent in mothers as compared with fathers, given results from previous studies in Norwegian [31,73] and that mothers, overall, likely would have spent more time with the infant, although less between 9 and 12 months of age, as typically this is when the fathers take the paternity leave (cf. details in §2). Our predictions and a brief introduction to all acoustic measures analysed in the current study can be found in table 1.

## 2. Methods

### 2.1. Participants

To recruit families to the current study, we sent postal leaflets to all parents residing in the greater Oslo region who had 6-month-old infants between May and July 2021 (450 in total). Seventy-one families agreed to participate, i.e. a ~15% response rate, which is expected based on previous recruitment rates in our laboratory. To be included in the study, our criteria were that (i) the infant was born full term (gestational weeks >37), (ii) the infant had no known visual or auditory impairments, (iii) the infant was exposed to 90% Norwegian or more at home, and (iv) both parents spoke Norwegian to the infant. Two families did not meet these criteria, both reporting that their infant received less than 90% exposure to Norwegian at home. As such, the final sample comprised *n* = 69 families.

Some attrition and missed sessions occurred over the five sessions the study comprised, running over 1 year, with *n* = 68 families participating in the T1 session (infant age 6 months), *n* = 67 at T2 (infant age 9 months), *n* = 62 at T3 (infant age 12 months), *n* = 61 at T4 (infant age 15 months) and *n* = 60 at T5 (infant age 18 months). The total attrition rate from the study onset to the study offset was thus 13%. Withdrawal from the study was attributed to parents not willing to come again to the laboratory due to time issues, and/or due to the family moving further away from the laboratory, making the visits less practical. In what follows, we report the demographics of both mothers and fathers from all 69 participating families. Note that for each session, only one parent (mother or father), who was the main caregiver at that time, came to the laboratory with the infant (see §2.2). Table 2 depicts relevant information on each session-specific parent–infant dyads.

**Table 2.** Parent–infant dyad characteristics for each laboratory session (T1–T5).

	T1 (*n* = 68)	T2 (*n* = 66)	T3 (*n* = 62)	T4 (*n* = 61)	T5 (*n* = 60)
infant age in days					
mean (s.d.)	182 (8.32)	276 (6.95)	365 (9.92)	452 (8.34)	542 (6.81)
range	168−204	268−302	350−401	441−477	523−565
infant gender					
*n* (%) girls	42 (61.8%)	41 (62.1%)	38 (61.3%)	38 (62.3%)	37 (61.7%)
*n* (%) boys	26 (38.2%)	25 (37.9%)	24 (38.7%)	23 (37.7%)	23 (38.3%)
parent gender					
*n* (%) mothers	67 (98.5%)	26 (39.4%)	22 (35.5%)	44 (72.1%)	43 (71.7%)
*n* (%) fathers	1 (1.5%)	40 (60.6%)	40 (64.5%)	17 (27.9%)	17 (28.3%)
parent reported % input to the infant^a^					
mean (s.d.)	69.3 (8.15)	62.4 (13.4)	58.4 (12.6)	55.0 (8.93)	54.7 (7.80)
range	50−90	30−90	25−100	40−80	40−80

^a^ This row reflects the percentage of input the parent who came to the laboratory (and was recorded) reported to provide at the time compared with the other parent (who was not recorded).

All parents were native speakers of Norwegian and reported that their infants were exposed to, on average, 99.1% Norwegian at home (s.d. = 2.55, range = 90–100). The majority of parents (70.8%, *n* = 52 mothers, *n* = 45 fathers) spoke the Eastern Norwegian dialect, the other parents spoke the Western (18.2%, *n* = 9 mothers, *n* = 16 fathers), Northern (8.1%, *n* = 5 mothers, *n* = 6 fathers) or Central (2.9%, *n* = 3 mothers, *n* = 1 father) dialect, respectively. Forty-one families (59%) considered themselves mono-dialectal, i.e. the mother and father both spoke the same dialect.^4^ At T1, mothers were on average 33.4 years of age (s.d. = 3.13, range = 28–41), and fathers were on average 35.9 years of age (s.d. = 4.25, range = 27–46). The parents’ highest level of education ranged from secondary school (2.9%, *n* = 2 mothers, *n* = 2 fathers), some higher education (4.4%, *n* = 6 fathers), bachelor’s degree (25.4%, *n* = 13 mothers, *n* = 22 fathers), master’s degree (64.5%, *n* = 51 mothers, *n* = 38 fathers), to doctoral degree (2.9%, *n* = 3 mothers, *n* = 1 father).

The current study was conducted according to the guidelines laid in the Declaration of Helsinki, with written informed consent obtained from a parent or a guardian for a child before any assessment or data collection. The study has been approved by the Norwegian Centre for Research Data (ref. 506879), and the local ethical committee at the Department of Psychology, University of Oslo (ref. 13066519). The preregistration, data, stimuli and analysis script for the study are openly available at the Open Science Framework (OSF) project page (https://osf.io/mbuzs).

### 2.2. Procedure and stimuli

Data collection took place in the infant laboratory at the Departmentof Psychology, University of Oslo, in five sessions—every three months across 1 year, during which the child was 6–18 months of age. Data were collected by four different experimenters (three female, one male (the first author), all native speakers of Norwegian and experienced in infant research), with no systematic pattern of assignment of experimenters across sessions. Data collection started with T1 in the summer of 2021 (when infants were 6 months of age) and ended with T5 in the summer of 2022 (when infants were 18 months of age). When infants were 6, 9, 12, 15 and 18 months of age (T1–T5, respectively), participating families were contacted to schedule their next visit to the laboratory. During scheduling, we encouraged the parent who considered themselves to be the ‘main caregiver’ at the time of testing, either the mother or the father, to come to the laboratory. This was to increase the likelihood that we would capture IDS from the parent who, at the time, provided the most speech input to the infant. This resulted in session-specific samples of predominantly mothers at T1 and fathers at T2–T3, in line with the typical parental leave pattern in Norway, where mothers take their leave before fathers.^5^ At T4–T5, most families had completed their parental leave, and again, as they considered themselves the main caregiver (reflected in the input provided, see table 2), a majority of mothers came to the laboratory. Approximately three days, and no more than one week, prior to their visit, parents filled in an online questionnaire that included, among others, information on general demographics, the infants’ linguistic environment and the MacArthur-Bates Communicative Development Inventories [92].

Upon their arrival to the laboratory, parents and their infants were first (re-)acquainted with the laboratory environment and experimenters, and received information about the course of their visit. Parents were not made aware of the specific purpose of the study (other than its relation to language development), or which parts of their recorded speech were of interest to the researchers, until after they had completed the final recording sessions at T5. As the study was part of a larger project that included assessment of infants’ language development, in each laboratory visit, the recordings of IDS and ADS were preceded by a vowel discrimination eye-tracking task and followed by a word comprehension eye-tracking task. The duration of a laboratory visit typically ranged between 45 and 60 min, with care being taken to accommodate the infants’ needs and readiness to engage in the tasks.

In all sessions, both IDS and ADS were elicited from the parent through reading a child-friendly picture book, specifically created for the purpose of the current study (and openly available for use; see OSF project page). A picture book was chosen over unstructured (e.g. [61]) and semi-structured (e.g. [54]) interactions, to rigorously control for the quantity and the quality of the linguistic context across registers, ages and speakers [76,77,79]. Book reading has been used in several studies on IDS (e.g. [66,71,72,93,94]), with similar results to studies using less-structured interactions (although effect sizes might be more conservative for intonational measures and more liberal for speaking rate [3].

**Figure 1.**
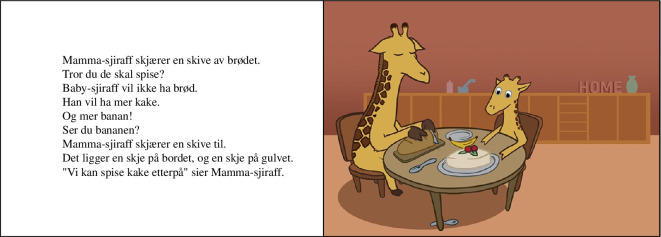
Example of one double-page in the picture book. Note that an English translation of the text is shown in table 3.

The picture book was printed in a hard-cover 19 × 15 cm^2^ format, written in Norwegian Bokmål,^6^ and contained five double pages, 48 sentences (counting interjections) and 220 words. Each double-page had a colourful illustration (created with Photoshop CC 2020) on the right side and a related short child-friendly narrative on the left side (figure 1; table 3). The narratives were not connected with each other, and their main protagonists were a bear, a train, a giraffe, a ball and a cow, respectively. In the current study, we analysed the production of nine Norwegian long (acoustically more salient than short) vowels /α:/, /e:/, /i:/, /u:/, /ʉ:/, /y:/, /æ:/, /ø:/ and /ɔ:/. In contrast to previous studies that used one word per vowel (e.g. sheep, shark, shoe for /i/, /α/, /u/), we assessed vowel production across five phonetic contexts, exemplified by five different words repeated twice over the course of the book, for a total of 90 target vowels. The words were mono- or bi-syllabic lexical and function words, counterbalanced in terms of their position within a sentence, such that each target vowel was present in at least one start-, mid- and end-sentence word. The target vowel was in a stressed position within the word, and, for the bi-syllabic words, with two exceptions, the target vowel was always placed in the first syllable. See appendix 1 for an overview of target vowels within words.

**Table 3.** Example of text from one page in the picture book (words with target vowels in bold, target vowels in italics, IPA transcripts in brackets).

original	English translation
mamma-sjiraff **skj*æ*rer** [ʂæ:rer] en **sk*i*ve** [ʂi:və] av **br*ø*det** [brø:ə]. Tror du de skal **sp*i*se** [spi:se]? Baby-sjiraff vil ikke ha **br*ø*d** [brø:]. Han vil ha **m*e*r** [me:r] **k*a*ke** [kα:kə]. Og **m*e*r** [me:r] **ban*a*n** [bαnα:n]! Ser du **ban*a*nen** [bαnα:nen]? Mamma-sjiraff **skj*æ*rer** [ʂæ:rer] en **sk*i*ve** [ʂi:və] til. Det ligger en **skj*e*** [ʂe:] på bordet, og en **skj*e*** [ʂe:] på gulvet. ‘**V*i*** [vi:] kan **sp*i*se** [spi:se] **k*a*ke** [kα:kə] etterpå,’ sier Mamma-sjiraff.	mommy-giraffe **cuts** a **slice** of **bread**. Do you think they’re going to **eat**? Baby-giraffe doesn’t want **bread**. He wants **more cake**. And **more banana**! Do you see the **banana**? Mommy-giraffe **cuts** another **slice**. There is a **spoon** on the table, and a **spoon** on the floor. ‘We can **eat cake** later,’ says Mommy-giraffe.

IDS and ADS recordings took place in the reception area in the laboratory, an 8 × 3 m^2^ room furnished in a child-friendly manner. During the IDS recording, the parent, sitting on a couch, read the picture book with their infant either on their lap or next to them on the couch (T2–T5 sessions) or next to them in a bouncing chair (T1 session). Parents were instructed to read and interact with their infant as they would typically do in a shared book-reading situation at home. The experimenter was not present in the room during the IDS recording. During the ADS recording, parents read the same picture book to an (adult) experimenter, with no further instructions but to read the book naturally as if reading to an adult. The experimenter was instructed to act naturally with smiles/nodding/eye-contact during the reading, but not give any verbal expressions. Notably, during the ADS recording, a second experimenter cared for the infant in an adjacent room, to avoid distracting the parent. A small section of the wall between the two rooms was cut-out glass, hence the parents were still able to see their infant if they wished to.

The order of the recordings was counterbalanced; half of the families started with the IDS recording, and the other half started with the ADS recording. The order within families was held constant across all sessions; i.e. for the parents of a given child, we always recorded ADS and IDS in the same order. We did so to reduce the complexity of the random effects structure in our models (which, otherwise, would also need to include the order of registers and its potential interactions in models that was already of considerable complexity). All sessions were recorded with a Zoom H4n handheld recorder in 44.1 kHz/16-bit WAV format. The recorder was placed on a boom microphone stand approx. 60 cm away from, and at the level of, the parents’ mouth, with the two microphones set to 90°, and the input level set to float between −12 and −6 dB to avoid clipping. At the end of each visit, parents were able to choose a small gift for their infant (e.g. a toy or a picture book, worth approx. 20 euros), have a polaroid of themselves and their infant taken and were reimbursed for any travel costs.

### 2.3. Data processing and acoustic measures

#### 2.3.1. Pre-processing

The IDS and ADS recordings (*n* = 635 in total) were processed in several steps to obtain the phrase- and vowel-level data to be used for the analyses. To accommodate for deviations from the picture book (e.g. repetitions), two trained research assistants first manually transcribed all audio recordings, before an automatic forced alignment procedure, the Montreal Forced Aligner [95], aligned the transcriptions with the audio on a phoneme-by-phoneme basis. The forced aligner requires a pre-trained acoustic model and a pronunciation dictionary specific to the relevant language. For our purposes, we utilized the acoustic model NoFA [96], tailored for Norwegian Bokmål and the pronunciation dictionary provided by the National Library of Norway. NoFA’s training is based on speech data sourced from the Language Bank’s NB Tale dictionary [97] and the phonetic section of the RUNDKAST database [98] and is optimized for use with the Montreal Forced Aligner. The output of the alignment procedure was 635 unique textgrid files containing two tiers: one with phonemes and other with words.

To obtain target vowels, two trained research assistants inspected the precision of the alignment (that occurred in 10 ms steps) of these specific vowels in the textgrid files using Praat [99], and manually adjusted their boundaries if necessary, following the same vowel onset and offset boundary definition as in Cristia & Seidl [69]. In total, *n* = 54 954 target vowels were identified, out of which *n* = 32 411 (59.4%) were manually adjusted. A customized Praat script [100] was run to collect duration (in ms) and the mean F1 and F2 (in Hz) of the target vowels, with pre-specified formant ceiling values at 5500 Hz for mothers and 5000 Hz for fathers.^7^ See appendix 2a for an overview of vowel data, and appendix 2b for a comparison of manually and non-manually adjusted vowels (indicating no marked differences).

We defined a phrase as a portion of continuous speech enclosed by a minimum 500 ms of silence. To obtain phrases, we used an automatic procedure in R [101], leveraging on the word tier of the textgrids generated by the forced alignment. By using the function ‘tier_to_df’ available in the R package *phonfieldwork* (version 0.0.11 [102]), we converted the word tier into a structured R dataframe. The word tier contained word segments along with the pauses between adjacent words. We systematically analysed each word, identifying and merging words into a single phrase if the pauses between them were shorter than 500 ms (as used in, e.g. [103]). Conversely, a new phrase began when pauses reached or exceeded the 500 ms threshold. This iterative process allowed us to create distinct phrases based on the temporal arrangement of pauses within the word tier. Consequently, the length and content of each phrase could vary, encompassing anything from short utterances to complete sentences. In total, we identified *n* = 22 958 phrases. A customized Praat script [100] was used to extract the duration (in ms), and minimum, maximum and mean F0 (in Hz) for each phrase.

To extract the number of syllables and phonation time of a phrase, needed for our measure of articulation rate, we used an approach implemented in Python [78]. The library *Praat-textgrids* [104] was used to count the number of vowels within a phrase, our index of number of syllables, while the library *librosa* [105] was used to compute the phonation time, i.e. the duration of a phrase that contained speech. Phonation time was calculated by analysing the audio waveform of each phrase to determine the duration of voiced segments present within it. This process comprises two steps that play distinct roles in identifying voiced segments where speech occurs and unvoiced segments representing silences within the audio of a corresponding phrase. First, the segmentation of the waveform is carried out using the ‘librosa.effects.split’ function. This function dissects the waveform into smaller segments based on energy levels. The ‘top_db’ parameter, set to a value of 20 dB, specifies the maximum allowable difference between the peak energy and energy in silent regions. This step effectively partitions the audio into candidate segments that could contain speech or silence. Second, determining whether a segment is voiced or unvoiced for each segmented portion of the audio, the root mean square energy, a measure of sound intensity, is calculated using the ‘librosa.feature.rms’ function. A threshold value is established through the combination of a decibel level (−25 dB) and the 0.99 quantiles of the waveform’s amplitude. By calculating the root mean square energy of each segment and comparing it with the threshold, voiced segments are identified as regions with energy surpassing the threshold, signifying speech presence. The cumulative durations of these voiced segments are the computed phonation time, quantifying the actual speech time span within a phrase.

#### 2.3.2. Acoustic measures

Vowel-level data were used to compute the following measures: vowel duration, vowel space areas (a corner version using /i:/-/æ:/-/u:/, and a full version using all border vowels /i:/-/e:/-/æ:/-/α:/-/ɔ:/-/u:/-/ʉ:/), vowel variability and vowel distinctiveness. Phrase-level data were used to compute the following measures: pitch, pitch range and articulation rate. Computation of the acoustic measures largely followed methods detailed in previous work [31,73] and are described briefly below and visualized in appendices 3−7.

*Vowel duration* is the full duration of a target vowel (in ms).

*Vowel space areas* are the overall size of the F1–F2 vowel space (in Hz^2^), for each participant, register and session, computed by the average F1 and F2 (in Hz) for each vowel category using the following formula (exemplified with three vowels, where ‘ABS’ is the absolute value): ABS One-half × [(F1/vowel_1_/ × (F2/vowel_2_/ – F2/vowel_3_/) + F1/vowel_2_/ × (F2/vowel_3_/ – F2/vowel_1_/) + F1/vowel_3_/ × (F2/vowel_1_/ – F2/vowel_2_/)] and so forth. We computed two versions of the vowel space area, one using the three most peripheral corner vowels /i:/-**/**æ:/-/u:/ (note that /æ:/, not /α:/, is the most peripheral vowel in Norwegian F1–F2 space), and one including all border vowels, namely /i:/-/e:/-/æ:/-/α:/-/ɔ:/-/u:/-/ʉ:/, as this measures the total vowel area most accurately as the actual vowel space may not necessarily correspond to a simple triangle.

*Vowel variability* is an index of the within-category precision in vowel production, measured by fitting F1 and F2 of all vowel tokens, exemplifying the category, to a customized MatLab script [106] which calculated the area of an ellipse (Hz^2^), adjusting for its position in the acoustic space, for each vowel category, participant, register and session, with the following formula: *σ*F1 *× σ*F2 *× π*, where *σ*F1 is the s.d. of F1, and *σ*F2 is the s.d. of F2. A high vowel variability score will indicate more variable and loose vowel categories, whereas a low vowel variability score will indicate more compact and precise vowel categories.

*Vowel distinctiveness* is an index of the proportion of variance in F1 and F2 explained by vowel category identity, computed as the between-vowel category sum of squares (the squared distances of category cluster centroids from the overall vowel space centroid) divided by the total sum of squares (squared distances of individual vowel tokens from the overall vowel space centroid), for each participant, register and session, for eight vowel categories (we omitted the category /y:/, as it fully overlaps with the Norwegian /i:/ in the F1–F2 space). The measure ranges from 0 (cluster membership does not explain any variance) to 1 (cluster membership explains all variance). Thus, while vowel variability indicates the precision of vowel production within each category, vowel distinctiveness indicates the discriminability of the categories, i.e. the degree of overlap, taking into account their distribution within the full vowel space.

*Pitch* is the mean pitch in a phrase. As pitch perception follows a logarithmic scale, Hz values were converted to semitones using the following formula: 12 × log^2^(F0/constant), with 10 as a constant (i.e. semitones-above-10-Hz).

*Pitch range* is the difference between the highest and lowest pitch values (in semitones, see above) in a phrase.

*Articulation rate* is the number of syllables in a phrase controlled for the phonation time of the phrase, i.e. the duration of the phrase after excluding any silences. In other words, the measure captures the speed at which syllables are articulated within a phrase, while considering the duration of the phrase itself, without including pauses or breaks.

### 2.4. Statistical analyses

To test our hypotheses regarding the differences between IDS and ADS registers, as well as the potential change in these differences across infants’ age and between mothers and fathers (see table 1), we fitted a set of mixed models in which the response variable was a given acoustic measure (pitch, pitch range, articulation rate, vowel duration, full and corner versions of vowel spaces, vowel variability and vowel distinctiveness), and the key predictor variables were register, infants’ age, parents’ gender and their interactions, in part also with other predictors. The specific fitting procedures are detailed for each acoustic measure in the following sections, and the results outlined in §3. All analyses were preregistered (https://osf.io/mbuzs) and conducted in R (version 4.3.1 [101]).

#### 2.4.1. Pitch (model 1)

To estimate the effects of register, child age and parent gender on pitch, we used a linear mixed model (LMM) [107], with register (IDS or ADS), child age (in days), parent gender (mother or father) and their interactions (up to order three) as fixed effects. To control for their potential effects, we also included phrase onset (time in the recording when the pitch value was taken),^8^ child gender (boy or girl) and recording order (IDS or ADS recorded first) as fixed effects, as well as the interactions between child and parent gender, and two-way interactions between phrase onset and the target fixed effects. To account for the variability within and between individual infants, parents and sessions, these were included as random intercept effects (session was nested in child). To avoid a model being overconfident with regard to the precision of estimates of fixed effects and interactions, as well as to keep type-I error rate at the nominal level of 5%, we included all theoretically identifiable random slopes and the correlations among random intercepts and slopes [108]. We considered the random slope of a fixed effects factor (e.g. ADS–IDS) within a grouping factor (e.g. child ID) as theoretically identifiable when for at least half of the levels of the grouping factor we had at least two data points per each level of the fixed effects factor. We considered the random slope of a fixed effects covariate (e.g. child age) as theoretically identifiable when we had either at least three unique values of the covariate for at least half the levels of the grouping factor or when we had at least two unique values of the covariate, each with at least two observations, for at least half the levels of the grouping factor. We applied these rules as these are obviously the minimum requirement for the model to be able to estimate the contribution of the random effects and residual s.d. (in Gaussian models, see also preregistration at https://osf.io/mbuzs. The full model structure is depicted in appendix 8a.

As an overall test of the fixed effects of register, and the interactions involving register, we conducted a full-null model comparison [90], aiming at avoiding cryptic multiple testing, whereby the null model lacked register and any interactions involving register, but was otherwise identical to the full model, including its random effects structure. The sample analysed with this model comprised a total of 22 958 pitch values (9045 in ADS; 13 913 in IDS), produced by 124 parents (68 mothers; 56 fathers) of 69 infants, in a total of 317 sessions.

#### 2.4.2. Pitch range (model 2)

To estimate the effects of register, child age and parent gender on pitch range, we used an LMM.^9^ The model structure and fitting procedure were identical to that of pitch, and as such, for brevity, we refer readers to that description. The sample analysed with this model was of the same size and structure as for model 1.

#### 2.4.3. Articulation rate (model 3)

To estimate the effects of register, child age and parent gender on articulation rate, we used a generalized linear mixed model (GLMM) [107], with Poisson error structure and log link function (originally we aimed for a zero-truncated model, but these failed to converge). The fixed effects and interaction terms were identical to the models of pitch and pitch range, with the addition of log-transformed phonation time (length of a phrase minus silences) as an offset term (log-transformed [109]). As such, instead of computing articulation rate (number of syllables per phonation time) prior to modelling and using this as the response variable, we used the number of syllables in a phrase as the response and controlled for phonation time by including it as an offset term in the model. The random effects structure of the model was initially identical to the models with pitch or pitch range being the response; i.e. random intercepts of children, parents and sessions and a maximal random slopes structure, including correlation parameters. However, as the model failed to converge (indicated by negative likelihood ratio test statistics resulting from dropping individual fixed effects one at a time and comparing reduced and full models), we simplified the model by removing the correlation estimates from the random effects structure. This led to an only minor decrease in model fit (log-likelihoods; full model including the correlation parameters = −50 016, d.f. = 108; full model lacking the correlation parameters = −50 030, d.f. = 36). To test the overall effect of register on articulation rate, we conducted a full-null model comparison whereby the null model lacked register and any interactions in it were involved in the fixed effects part. The model structure is depicted in appendix 10a. The sample analysed with this model was of the same size and structure as for models 1 and 2.

#### 2.4.4. Vowel duration (model 4)

To estimate the effects of register, child age and parent gender on vowel duration, we used an LMM. With the exception of removing phrase onset, the fixed effects and interaction terms were identical to the above models on pitch and pitch range. The random effects structure included random intercepts for child, parent and session, but also word (which word the vowel duration was sampled from) and vowel (which vowel category the duration was sampled from). The random slopes structure mirrored the fixed effects structure in vowel and word, whereas we included random slopes of child age, register and their interaction within parent and child and an additional random slope of child age within child. Initially, the random effects structure also included correlation parameters between all random intercepts and slopes. However, a ‘singular fit’ message suggested some of the random effects terms to be unidentifiable. Hence, we simplified the model by removing the correlation estimates from the random effect of parent. This led to an only minor decrease in model fit (log-likelihoods, full model including the correlation parameters = −11 793, d.f. = 172; full model lacking the correlation parameters = −11 794, d.f. = 166). The model is outlined in appendix 11a. To assess the overall effect of register on vowel duration, we conducted a full-null model comparison whereby the null model lacked register and any interactions in it were involved in the fixed effects part. The sample analysed with this model comprised a total of 54 954 vowel duration values (27 862 in ADS; 27 092 in IDS), taken from 9 vowel categories in 45 words, produced by 124 parents (68 mothers; 56 fathers) of 69 infants, in a total of 317 sessions.

#### 2.4.5. Vowel space area corner version (model 5)

To estimate the effects of register, child age and parent gender on vowel space area (using corner vowels /i:/-/æ:/-/u:/), we used an LMM. The fixed effects and interaction terms were identical to the model on vowel duration. The random effects structure included random intercepts of child, parent and session, and random slopes of child age, register, parent gender and the interactions between child age on the one hand and register and parent gender on the other in child, and those of child age and register within parent. Initially, the random structure included parameters for correlations among random intercepts and slopes. However, a ‘singular fit’ message suggested some of the random effects terms to be unidentifiable. Hence, we simplified the model by removing all correlation estimates from the random effects within parent. This led to an only minor decrease in model fit (log-likelihoods; full model including the correlation parameters = −7557, d.f. = 40; full model lacking the correlation parameters = −7560, d.f. = 37). The model is outlined in appendix 12a. We conducted a full-null model comparison whereby the null model lacked register and all interactions in it were involved in the fixed effects part. The sample analysed with this model comprised a total of 614 vowel space (corners) values (307 in ADS; 307 in IDS), taken from 124 parents (68 mothers; 56 fathers) of 69 infants, in a total of 308 sessions.

#### 2.4.6. Vowel space area full version (model 6)

To estimate the effects of register, child age and parent gender on vowel space area (full version using /i:/-/e:/-/æ:/-/α:/-/ɔ:/-/u:/-/ʉ:/), we used an LMM. The model structure and fitting procedure were identical to that of the corner version of the vowel space as outlined above, and as such, for brevity, we refer readers to that description. As for the corner version model, we removed the correlation estimates in the random effects structure within parent, as these appeared unidentifiable. Log-likelihoods suggested only a small decrease in model fit (full model including the correlation parameters = −7631, d.f. = 40; full model lacking the correlation parameters = −7633, d.f. = 37). We conducted a full-null model comparison whereby the null model lacked register and all interactions in it were involved in the fixed effects part. The sample analysed with this model was of the same size and structure as for model 5.

#### 2.4.7. Vowel variability (model 7)

To estimate the effects of register, child age and parent gender on vowel variability, we fitted an LMM. The fixed effects and interaction terms were identical to those in the models of vowel duration and vowel spaces. The random structure included random intercepts of child, parent, session and vowel, and random slopes of register, child age, parent gender within child, register, child age and their interaction within parent, register within session, and register, child age, parent gender and all their interactions up to order three, as well as child gender and recording order within vowel and also all parameters for correlations among random intercepts and slopes. The model structure is outlined in appendix 14a. We conducted a full-null model comparison whereby the null model lacked register and any interactions in it were involved in the fixed effects part. The sample analysed with this model comprised a total of 5658 vowel variability values (2835 in ADS; 2823 in IDS), taken from 9 vowel categories, from 124 parents (68 mothers; 56 fathers) of 69 infants, in a total of 317 sessions.

#### 2.4.8. Vowel distinctiveness (model 8)

To estimate the effects of register, child age and parent gender on vowel distinctiveness, we used a GLMM, with beta error distribution and logit link function [110]. The fixed effects and interaction terms were identical to those in the models on vowel duration, vowel spaces and vowel variability. The random effects structure of the model included random intercepts of children, parents and sessions, random slopes of age, register and parent gender within child and register within parent, without interactions or correlation parameters. This reduced random structure was a result of several of the initial models not converging. The model structure is outlined in appendix 15a. To test the overall effect of register on vowel distinctiveness, we conducted a full-null model comparison whereby the null model lacked register and any interactions in it were involved in the fixed effects part. The sample analysed with this model was of the same size and structure as for model 6.

#### 2.4.9. Implementation

We fitted the models in R using the functions lmer (models 1, 2, 4, 5, 6 and 7) or glmer (model 3) of the package lme4 (version 1.1-34) [111] or the function glmmTMB of the equally named package [112] (model 8). Prior to fitting the models we inspected all quantitative predictors and in case of LMMs also the response for whether their distributions were roughly symmetric. When covariates were skewed we log-transformed them, and in case of LMMs, we also log-transformed the response when it was skewed (models 4 and 7). Note that vowel duration was rounded to the nearest 10 ms prior to log-transformation, to account for any subtle differences in manually adjusted and non-manually adjusted vowel durations. We then *z*-transformed covariates to a mean of zero and an s.d. of one to achieve an easier interpretable model [113] and ease model convergence. We estimated the significance of individual fixed effects in LMMs by means of the Satterthwaite approximation [114] using the function lmer of the package lmerTest (version 3.1-3 [115]); and a model fitted with restricted maximum likelihood. In the case of GLMMs (models 3 and 8), we tested the significance of individual fixed effects by dropping them from the model one at a time and comparing the likelihoods of the resulting reduced models with that of the full model (R function drop1). We based these tests, and also the full-null model comparisons, on likelihood ratio tests [116]. We estimated 95% confidence intervals of model estimates and fitted values by means of parametric bootstraps (*n* = 1000 bootstraps; function bootMer of the package lme4 for models 1–7; function simulate of the package glmmTMB for model 8).

In the case of a significant full-null model comparison, but non-significant (*p* > 0.1) interactions being present in the model, we iteratively removed them until all non-significant interactions had been removed (as specified in the preregistration). We did so in order to be able to infer about the significance of the respective lower order terms and to obtain unconditional estimates of effects [117]. Note that in a given step of this process, we removed all non-significant interactions of the same and highest order (e.g. all three-way interactions) at the same time. Note also that this iterative removal is different from step-wise model simplification as it (i) is conditional on a significant full-null model comparison, (ii) in a given step removes all non-significant interactions of the same (and the highest) order simultaneously, and (iii) considers only interactions but not main effects.

We assessed model stability on the level of the estimated coefficients by excluding the individual levels of the random effects (e.g. individual children and individual parents) one at a time [118], fitting the full model to each of the subsets and comparing the estimates obtained with those obtained for the full dataset. This revealed the fixed effects estimates of all models to be of good to excellent stability (see tables in the appendices). Note that in case of model 4, we could neither estimate confidence intervals nor assess model stability as doing so was computationally not feasible due to the size of the dataset (see below) and the complexity of the random effects structure. We assessed whether collinearity was an issue by means of variance inflation factors (VIF) [119], which we obtained for models lacking interactions in the fixed effects part and using the function vif of the package car [120] or the function check_collinearity of the package performance [121]. Collinearity appeared to be no issue (maximum VIF across all models: 1.25 [122]). For LMMs, we checked whether the assumptions of normally distributed and homogeneous residuals were met by visual inspection of a QQ-plot of residuals and residuals plotted against fitted values [119,122]. These were considered acceptable for model 1 and met for models 2, 5, 6 and 7 (see OSF project page). The residuals of model 4 seemed to be normally distributed but were clearly heteroscedastic. Hence the results of this model should be interpreted with caution. Neither in model 3 nor in model 8 was the response overdispersed given the model (dispersion parameter, model 3: 0.557; model 8: 0.735).

## 3. Results

The specific fitted model and results are detailed for each acoustic measure in the following sections. Table 4 depicts the by-participant mean, s.d., range and number of observations for each acoustic measure, grouped by gender, register and session.

**Table 4.** Mean, s.d., range and number of observations of each acoustic measure, grouped by parent gender, register and session (T1–T5).

		mothers’ ADS				mothers’ IDS				fathers’ ADS				fathers’ IDS			
session	acoustic measure	*M*	s.d.	range	*N*_obs_ (*N*_participants_)	*M*	s.d.	range	*N*_obs_ (*N*_participants_)	*M*	s.d.	range	*N*_obs_ (*N*_participants_)	*M*	s.d.	range	*N*_obs_ (*N*_participants_)
**T1**	pitch	52.6	2.61	37.8−61.8	1865 (67)	54.5	3.3	41.5−70.1	2605 (67)	44.1	0.94	42.3−46.4	32 (1)	46.1	1.99	42.3−51.2	48 (1)
	pitch range	12.64	3.78	1.45−24.86	1865 (67)	14.2	4.91	0.18−30.1	2605 (67)	7.71	2.31	3.55−11.9	32 (1)	11.81	4.26	2.36−17.8	48 (1)
	articulation rate	4.98	1.315	1.14−32.67	1865 (67)	4.65	1.382	0.44−13.2	2605 (67)	6.21	1.491	2.72−9.1	32 (1)	5.36	1.968	1.04−11.4	48 (1)
	vowel duration	127.5	42.04	30−433	5918 (67)	148.9	67.22	26−1173	5796 (67)	94.5	27.47	30−173	88 (1)	120.8	50.22	40−343	85 (1)
	vowel space corners	266.1	67.7	150−439.8	67 (67)	249.7	62.26	99.6−384	67 (67)	135.8	NA	136−136	1 (1)	206.4	NA	20−206	1 (1)
	vowel space full	336.6	79.48	211.6−522	67 (67)	326.1	71.71	160.7−493	67 (67)	209	NA	209−209	1 (1)	278.8	NA	279−279	1 (1)
	vowel variability	202.6	141.92	19.4−939.1	603 (67)	370.4	246.1	55.4−2007	603 (67)	162.9	72.52	63.5−296	9 (1)	288.4	202.4	85.4−742	9 (1)
	vowel dist.	0.87	0.04	0.75−0.95	67 (67)	0.8	0.08	0.55−0.92	67 (67)	0.81	NA	0.81−0.81	1 (1)	0.82	NA	0.82−0.82	1 (1)
**T2**	pitch	52	2.51	40−61.4	696 (26)	54.9	3.51	45.3−66.7	1123 (26)	42.8	2.95	34.7−52.7	1244 (38)	44.9	3.64	34.7−67.8	1761 (39)
	pitch range	12.98	3.96	0.2−28.35	696 (26)	13.61	5.06	0.06−28.04	1123 (26)	11.68	4.05	0.52−28.43	1244 (38)	13.16	5.2	0.15−38.71	1761 (39)
	articulation rate	5.1	1.527	0.76−27.78	696 (26)	4.78	1.451	0.84−12.5	1123 (26)	5.16	1.35	1.59−15.18	1244 (38)	4.88	1.598	0.54−27.08	1761 (39)
	vowel duration	123.8	44.14	30−348	2283 (26)	153.1	80.18	30−1396	2206 (26)	119.6	42.68	30−349	3338 (38)	135.1	59.49	30−700	3309 (39)
	vowel space corners	284.9	81.6	150.8−450	26 (26)	269.8	71.36	125.3−464.2	25 (25)	137.8	38.83	65.7−237.5	38 (38)	160.5	49.23	71.1−271	39 (39)
	vowel space full	352.4	99.17	215.1−639.7	26 (26)	354.2	78.21	217.2−549.2	25 (25)	197.3	51.6	94.1−307.9	38 (38)	223.5	57.08	129−379.7	39 (39)
	vowel variability	192.9	128.84	25.3−852.5	234 (26)	409.4	265.6	76−1805.2	232 (26)	140	134.08	15.1−779.7	342 (38)	213.1	185.3	21.6−1333.4	351 (39)
	vowel dist.	0.88	0.04	0.8−0.93	26 (26)	0.8	0.06	0.69−0.93	25 (25)	0.83	0.07	0.63−0.95	38 (38)	0.81	0.06	0.66−0.92	39 (39)
**T3**	pitch	52.2	2.6	41−59.3	566 (21)	54.8	3.23	45.4−65.3	867 (21)	43.2	3.12	32.9−55.2	1251 (41)	45.1	4.02	33.7−62.3	1807 (41)
	pitch range	14.2	5.3	1.25−31.98	566 (21)	13.88	5.17	0.2−26.97	867 (21)	11.69	3.34	0−20.74	1251 (41)	13.22	4.63	0−25.67	1807 (41)
	articulation rate	5.15	1.366	0.46−18.47	566 (21)	4.77	1.778	0.51−24.51	867 (21)	5.17	1.38	1.56−23.44	1251 (41)	4.92	1.642	0.54−27.78	1807 (41)
	vowel duration	117.4	39.9	30−301	1862 (21)	146.3	74.11	30−1094	1787 (21)	117.7	39.41	30−338	3632 (41)	135	58.39	29−645	3445 (41)
	vowel space corners	246.7	58.74	177.5−378.2	21 (21)	256.9	73.86	136−421.6	21 (21)	143.1	36.14	75−207.5	41 (41)	154	62.68	51.6−285.5	38 (38)
	vowel space full	297	61.54	186.5−441	21 (21)	311.8	80.12	167.5−429.2	21 (21)	200.2	45.83	100.2−272.2	41 (41)	225.9	85.76	76.7−423.9	38 (38)
	vowel variability	188.7	123.33	33.1−1023.2	189 (21)	429.4	287.9	47.5−1793.2	189 (21)	115.2	90.25	11.6−576.3	369 (41)	203.6	162.8	7.4−1066.7	361 (41)
	vowel dist.	0.87	0.04	0.79−0.93	21 (21)	0.78	0.09	0.6−0.92	21 (21)	0.85	0.06	0.71−0.94	41 (41)	0.8	0.07	0.55−0.91	38 (38)
**T4**	pitch	52	2.4	39.9−61	1214 (43)	54.8	3.52	44.9−70.5	2130 (43)	43.3	3.11	37−53.4	474 (18)	45.4	3.82	35.1−61.5	759 (18)
	pitch range	13.17	4.25	0.64−27.86	1214 (43)	14.46	5.25	0.07−27.53	2130 (43)	10.82	3.65	0.96−18.86	474 (18)	13.75	4.81	0−27.83	759 (18)
	articulation rate	5.26	1.842	1.21−27.24	1214 (43)	4.81	1.658	0.32−23.44	2130 (43)	5.56	2.261	1.59−40.87	474 (18)	5.11	1.495	0.85−12.71	759 (18)
	vowel duration	120.9	41.36	25−343	3812 (43)	154.5	75.6	30−808	3691 (43)	114.8	37.97	39−310	1588 (18)	129.1	53.62	30−587	1479 (18)
	vowel space corners	260.7	72.79	123.6−487	43 (43)	266.3	75.55	64.9−417.6	42 (42)	139.2	45.29	78.9−210.5	18 (18)	164.2	85.47	61.3−342.9	16 (16)
	vowel space full	314.8	76.16	179.9−580.3	43 (43)	333.3	94.65	132.3−491.5	42 (42)	193.9	59.08	114.1−306	18 (18)	230.1	91.1	82.2−403.4	16 (16)
	vowel variability	187.7	146.69	35.3−1361.8	387 (43)	396.5	261.3	68.7−2034.1	386 (43)	116.8	108.18	28−823.2	162 (18)	207.7	153.6	30.7−922.1	159 (18)
	vowel dist.	0.87	0.05	0.75−0.94	43 (43)	0.79	0.07	0.65−0.89	42 (42)	0.84	0.08	0.55−0.92	18 (18)	0.82	0.07	0.67−0.92	16 (16)
**T5**	pitch	51.7	2.77	41.5−60.4	1203 (42)	54.9	3.73	44.4−68.2	1942 (41)	42.8	2.98	34.9−52.1	500 (18)	44.8	3.37	35.3−56.8	871 (19)
	pitch range	12.95	4.3	1.82−28.12	1203 (42)	15.07	5.21	0.12−27.59	1942 (41)	11.58	3.8	1.21−21.83	500 (18)	13.27	4.74	0.57−30.81	871 (19)
	articulation rate	5.23	1.597	0.69−22.5	1203 (42)	4.79	1.692	0.34−24.19	1942 (41)	5.64	2.066	1.67−23.19	500 (18)	4.9	1.657	0.69−25.86	871 (19)
	vowel duration	113	42.02	24−669	3767 (42)	145.6	79.58	27−760	3616 (41)	105.7	40	24−350	1574 (18)	128	61.53	19−545	1678 (19)
	vowel space corners	275.8	79.63	83−480.8	42 (42)	292.3	56.82	173.4−412.6	40 (40)	147.2	43.17	58.7−222.3	18 (18)	193.4	69.38	60−323.3	19 (19)
	vowel space full	329	90.1	100.4−497	42 (42)	352.5	69.69	200.8−509.7	40 (40)	206.3	44.58	102.8−285.6	18 (18)	254.8	85.56	103.9−440.2	19 (19)
	vowel variability	198.9	149.92	26.7−1155.8	378 (42)	362.9	227.3	38.1−1855.3	362 (41)	124.2	116.48	13.3−800.2	162 (18)	184.7	173.4	14.9−1187.6	171 (19)
	vowel dist.	0.88	0.05	0.71−0.94	42 (42)	0.82	0.06	0.69−0.93	40 (40)	0.84	0.08	0.65−0.94	18 (18)	0.83	0.09	0.67−0.93	19 (19)

Pitch and pitch range are in semitones, articulation rate in syllables per seconds phonation time, vowel duration in milliseconds, vowel space areas and vowel variability in kHz^2^ and vowel distinctiveness in quotients.

### 3.1. Pitch (model 1)

**Figure 2.**
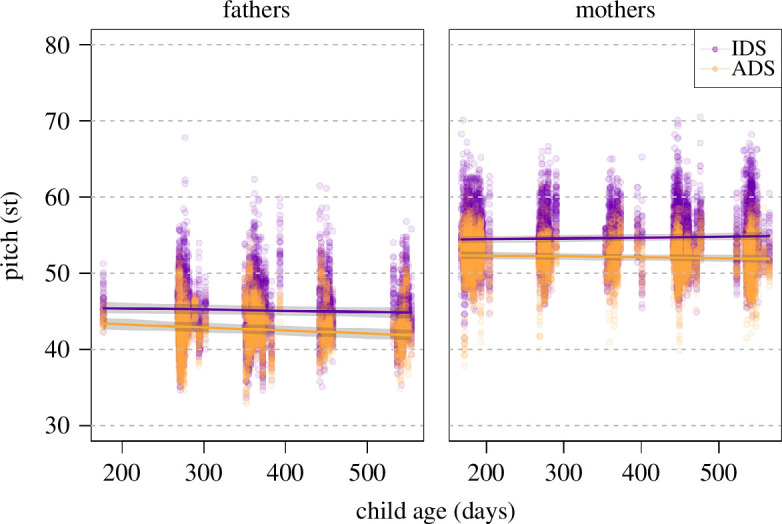
Pitch as a function of register and child age. Coloured lines show the fitted final model and the shaded areas its 95% confidence interval for all other predictors being at their average (model 1).

Overall, register (and its interactions, see below) had a clear impact on pitch, as indicated by a likelihood ratio test comparing the full and null model (*χ*^2^ = 117.1, d.f. = 9, *p* < 0.001). After the removal of all non-significant interactions (appendices 8b and 8c), we found that parents’ pitch in ADS decreased with child age, while pitch remained relatively stable in IDS (significant interaction; table 5; figure 2). Given the fundamental differences in pitch between men and women, figure 2 depicts the interaction separately for mothers and fathers (but note that the three-way interactions with parent gender were not significant, and a collapsed plot is provided in appendix 8d). Furthermore, the reduced model indicated that pitch in ADS decreased with later phrase onset, while it remained relatively stable in IDS (significant interaction; table 5; appendix 8e). As can be seen in figure 2, IDS was overall expressed with higher pitch than ADS. The estimated results for the random effects in the full model are in appendix 8f.

**Table 5.** Results of the reduced model (without the non-significant three-way and two-way interactions) with pitch (in semitones) as the response (model 1; estimates, together with s.e., confidence limits and significance tests).

term	estimate	s.e.	lower CI	upper CI	*t*	d.f.	*p*
intercept	42.796	0.399	41.974	43.579			
register^a^	2.497	0.156	2.151	2.803			
phrase onset^b^	−0.356	0.045	−0.448	−0.267			
child age^c^	−0.479	0.132	−0.710	−0.211			
parent gender^d^	9.508	0.379	8.752	10.237			
child gender^e^	0.061	0.329	−0.562	0.769	0.185	56.478	0.854
recording order^f^	−0.366	0.324	−0.983	0.353	−1.131	57.738	0.263
register:phrase onset	0.349	0.048	0.252	0.442	7.227	224.891	<0.001
register:child age	0.283	0.080	0.128	0.442	3.555	64.092	0.001
child age:parent gender	0.334	0.139	0.058	0.580	2.402	87.452	0.018

Note that for this and other tables, significance tests of fixed effects that are involved in significant interactions are not shown due to limited interpretability.

^a^ Dummy coded with ADS being the reference category.

^b^ *z*-transformed to a mean of 0 and an s.d. of 1, mean (s.d.) of the original variable were 69 275 (47 508) ms.

^c^ *z*-transformed to a mean of 0 and an s.d. of 1, mean (s.d.) of the original variable were 362 (126.7) days.

^d^ Dummy coded with father being the reference category.

^e^ Dummy coded with boy being the reference category.

^f^ Dummy coded with ADS–IDS being the reference category.

### 3.2. Pitch range (model 2)

**Figure 3.**
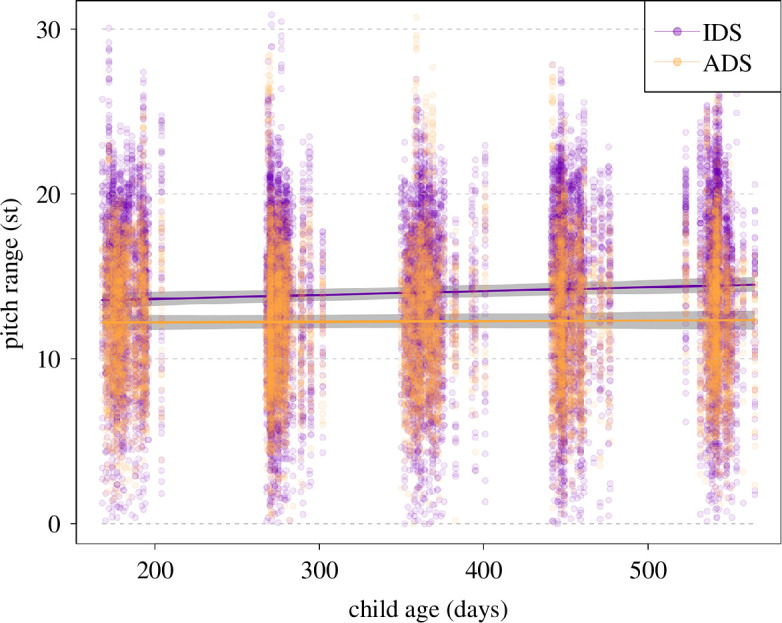
Pitch range as a function of register and child age. Coloured lines show the fitted model (model 2) and the shaded areas its 95% confidence interval for all other predictors being at their average, and the area of datapoints reflects number of observations, ranging from 1 to 12 (note that the *y*-axis was truncated to highlight the trend, and some individual points are not plotted; a non-truncated plot is shown in appendix 9e).

Overall, register (and its interactions, see below) had a clear impact on pitch range, as indicated by a likelihood ratio test comparing the full and null model (*χ*^2^ = 69.38, d.f. = 9, *p* < 0.001). After the removal of all non-significant interactions (appendices 9a and 9b), we found that parents’ pitch range in IDS increased with child age, while it remained relatively stable in ADS (significant interaction; table 6; figure 3). Furthermore, parents’ pitch range in ADS decreased with later phrase onset and more so than pitch range in IDS (significant interaction; table 6; appendix 9c). As can be seen in the plots, IDS was overall expressed with a wider pitch range than ADS. The estimated results for the random effects in the full model are depicted in appendix 9d.

**Table 6.** Results of the reduced model (lacking the non-significant three-way and two-way interactions) with pitch range (in semitones) as the response (model 2; estimates, together with s.e., confidence limits and significance tests).

term	estimate	s.e.	lower CI	upper CI	*t*	d.f.	*p*
intercept	11.818	0.356	11.117	12.514			
child age^a^	0.051	0.100	−0.152	0.263			
register^b^	1.744	0.171	1.402	2.104			
phrase onset^c^	−0.786	0.074	−0.920	−0.644			
parent gender^d^	1.135	0.286	0.539	1.719	3.969	58.616	<0.001
child gender^e^	−0.215	0.348	−0.904	0.463	−0.616	51.839	0.540
recording order^f^	−0.209	0.341	−0.907	0.522	−0.613	53.014	0.543
child age:register	0.249	0.118	0.027	0.496	2.117	94.240	0.037
child age:phrase onset	0.059	0.036	−0.010	0.132	1.622	121.489	0.107
register:phrase onset	0.327	0.082	0.156	0.475	3.988	243.095	<0.001

^a^ *z*-transformed to a mean of 0 and an s.d. of 1, mean (s.d.) of the original variable were 362 (126.7) day.

^b^ Dummy coded with ADS being the reference category.

^c^ *z*-transformed to a mean of 0 and an s.d. of 1, mean (s.d.) of the original variable were 69 275 (47 508) ms.

^d^ Dummy coded with father being the reference category.

^e^ Dummy coded with boy being the reference category.

^f^ Dummy coded with ADS–IDS being the reference category.

### 3.3. Articulation rate (model 3)

**Figure 4.**
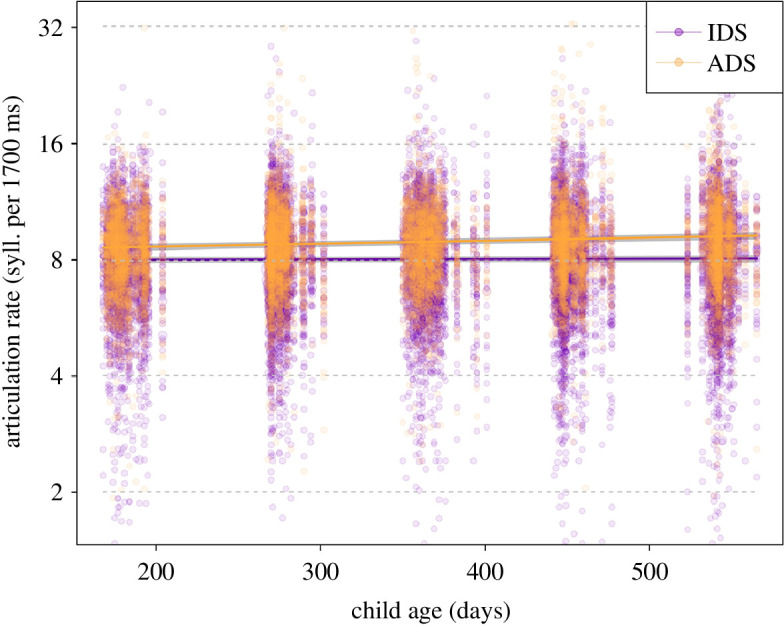
Articulation rate (number of syllables per 1700 ms phonation time) as a function of register and child age.^10^ Coloured lines show the fitted model (model 3) and the shaded areas its 95% confidence interval for all other predictors being at their average (note that the *y*-axis was truncated to highlight the trend, and some individual points are not plotted; a non-truncated plot is shown in appendix 10d).

Overall, register (and its interactions, see below) had a clear impact on articulation rate (i.e. number of syllables controlled for phonation time), as indicated by a likelihood ratio test comparing the full and null model (*χ*^2^ = 128.3, d.f. = 9, *p* < 0.001). After the removal of all non-significant interactions (appendices 10b and 10c), we found that parents’ articulation rate in ADS increased with child age, while it remained relatively stable in IDS (significant interaction; table 7; figure 4). As can be seen in the plot, IDS was overall expressed with lower articulation rate than ADS. The estimated results of the random effects of the full model are in appendix 10d.

### 3.4. Vowel duration (model 4)

**Figure 5.**
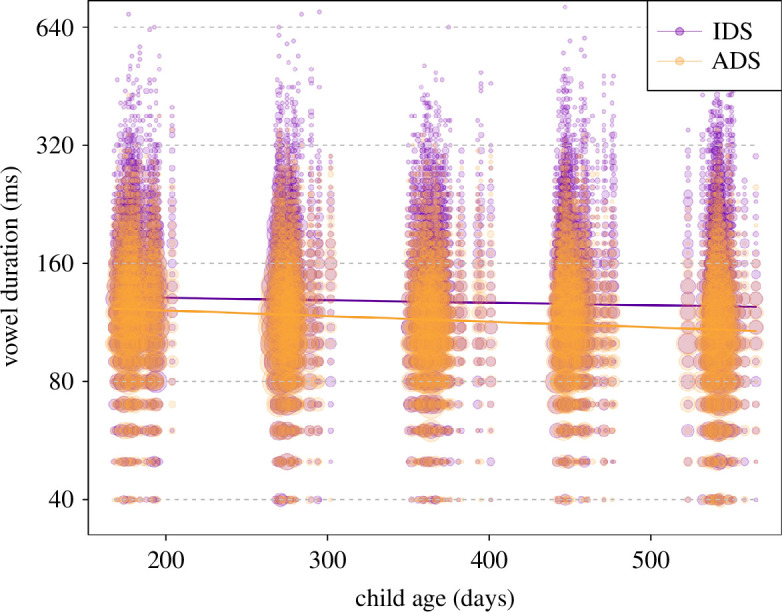
Vowel duration as a function of register and child age. Coloured lines show the fitted model (model 4) for all other predictors being at their average and the area of data points reflects the number of observations, ranging from 1 to 115 (note that the *y*-axis was truncated to highlight the trend, and some individual points are not plotted; a non-truncated plot is shown in appendix 11e).

**Figure 6.**
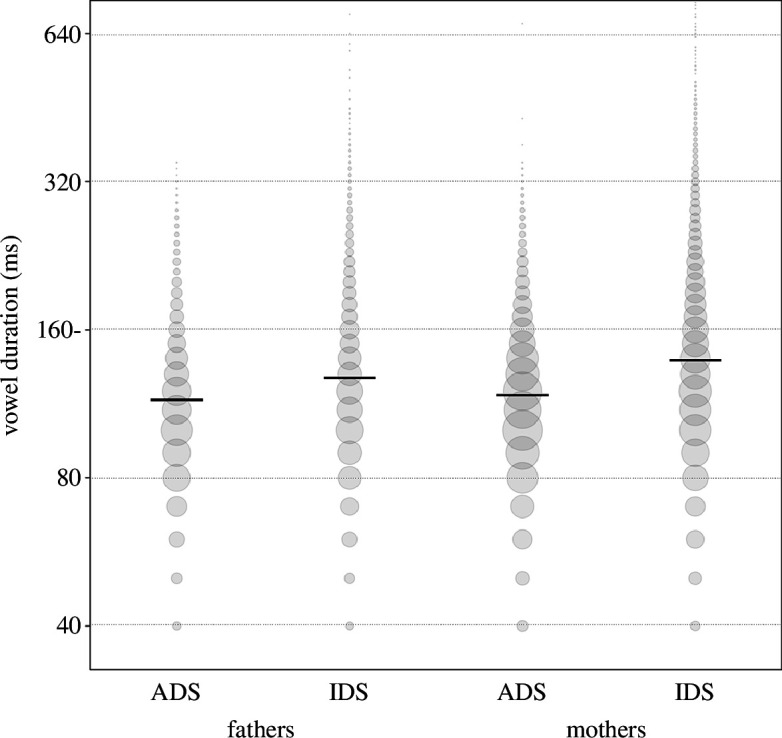
Vowel duration as a function of register and parent gender. Horizontal black lines show the fitted model (model 4) for all other predictors being at their average, and the area of data points reflects the number of observations, ranging from 1 to 2192 (note that the *y*-axis was truncated to highlight the trend, and some individual points are not plotted; a non-truncated plot is shown in appendix 11f).

Overall, register (and its interactions, see below) had a clear impact on vowel duration, as indicated by a likelihood ratio test comparing the full and null model (*χ*^2^ = 36.36, d.f. = 6, *p* < 0.001). After removal of all non-significant interactions (appendices 11b and 11c), we found that parents’ vowel duration decreased with child age, and more so in ADS than in IDS (significant interaction of register and child age; table 8; figure 5). Further, mothers increased their vowel duration more in IDS as compared with ADS than fathers did (significant interaction of register and parent gender; table 8; figure 6). As can be seen in the plots, IDS was overall expressed with longer vowel duration than ADS. The estimated results of the random effects of the full model are in appendix 11d.

### 3.5. Vowel space area (corner version; model 5)

**Figure 7.**
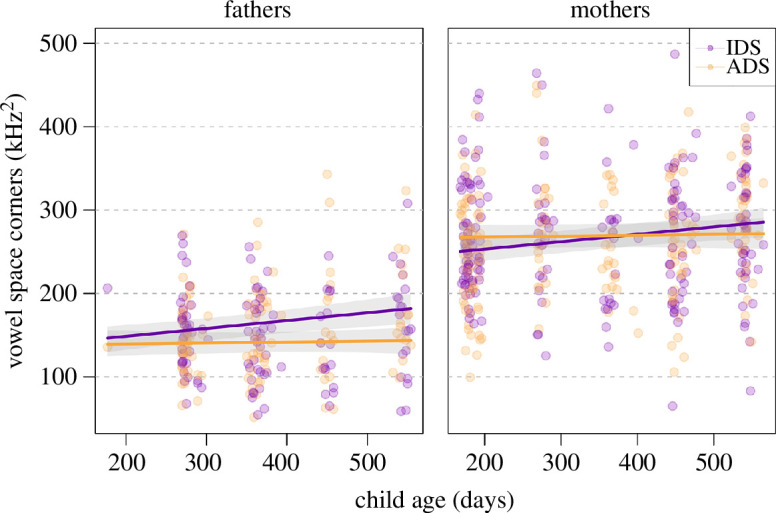
Vowel space area (corners) as a function of register and child age. Coloured lines show the fitted final model (model 5) and the shaded areas its 95% confidence interval for all other predictors being at their average.

**Figure 8.**
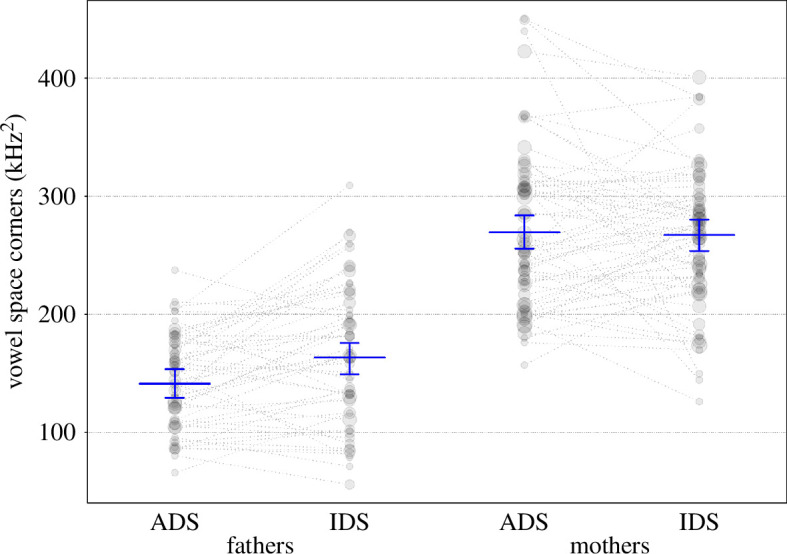
Vowel space area (corners) as a function of register and parent gender. Horizontal blue lines show the fitted model (model 5) and vertical lines its 95% confidence interval for all other predictors being at their average, dotted lines connect individual parents and the area of the data points reflects the number of observations, ranging from 1 to 5.

Overall, register (and its interactions, see below) had a clear impact on vowel space area (corner version using /i:/-/æ:/-/u:/), as indicated by a likelihood ratio test comparing the full and null model (*χ*^2^ = 16.28, d.f. = 4, *p* = 0.003). After the removal of all non-significant interactions (appendices 12b and 12c), we found that parents’ vowel space area (corners) slightly increased in IDS with child age, while it slightly decreased in ADS (significant interaction; table 9; figure 7). Given the fundamental difference in vowel space area between men and women, figure 7 depicts the interaction between age and register separately for mothers and fathers (but note that the three-way interactions with parent gender were not significant, and a collapsed plot is provided in appendix 12d). Furthermore, fathers’, but not mothers’, vowel space area (corners) was overall larger in IDS as compared with ADS (significant interaction; table 9; figure 8). The estimated results of the random effects of the full model are in appendix 12e.

**Table 7.** Results of the reduced model (lacking the non-significant three-way and two-way interactions) with articulation rate (in number of syllables controlled for phonation time) as the response (model 3; estimates, together with s.e., confidence limits and significance tests).

term	estimate	s.e.	lower CI	upper CI	*χ*2	d.f.	*p*
intercept	1.630	0.018	1.597	1.665			
child age^a^	0.023	0.004	0.015	0.031			
register^b^	−0.104	0.008	−0.120	−0.089			
phrase onset^c^	0.019	0.003	0.014	0.024	45.283	1	<0.001
parent gender^d^	0.012	0.018	−0.022	0.047			
child gender^e^	0.040	0.021	−0.002	0.080			
recording order^f^	0.019	0.016	−0.011	0.049	1.435	1	0.231
child age:register	−0.021	0.005	−0.030	−0.011	18.789	1	<0.001
parent gender:child gender	−0.043	0.023	−0.090	0.003	3.304	1	0.069

^a^ *z*-transformed to a mean of 0 and an s.d. of 1, mean (s.d.) of the original variable were 362 (126.7) day.

^b^ Dummy coded with ADS being the reference category.

^c^ *z*-transformed to a mean of 0 and an s.d. of 1, mean (s.d.) of the original variable were 69 275 (47 508) ms.

^d^ Dummy coded with father being the reference category.

^e^ Dummy coded with boy being the reference category.

^f^ Dummy coded with ADS–IDS being the reference category.

**Table 8.** Results of the reduced model (lacking the non-significant three-way and two-way interactions) with vowel duration (in log-transformed ms) as the response (model 4; estimates, together with s.e. and significance tests).

term	estimate	s.e.	*t*	d.f.	*p*
intercept	4.745	0.047			
register^a^	0.104	0.016			
child age^b^	−0.042	0.006			
parent gender^c^	0.021	0.018			
child gender^d^	−0.035	0.020	−1.767	62.713	0.082
recording order^e^	−0.062	0.019	−3.214	62.786	0.002
register:child age	0.023	0.007	3.489	21.802	0.002
register:parent gender	0.060	0.017	3.551	83.252	0.001

^a^ Dummy coded with ADS being the reference category.

^b^ *z*-transformed to a mean of 0 and an s.d. of 1, mean (s.d.) of the original variable were 358 (128.1) days.

^c^ Dummy coded with father being the reference category.

^d^ Dummy coded with boy being the reference category.

^e^ Dummy coded with ADS–IDS being the reference category.

**Table 9.** Results of the reduced model (lacking the non-significant three-way and two-way interactions) with vowel space area (corner version) (in Hz^2^) as the response (model 5; estimates, together with s.e., confidence limits and significance tests).

term	estimate	s.e.	lower CI	upper CI	*t*	d.f.	*p*
intercept	150 476	9313	131 768	168 181			
register^a^	22 055	7073	7321	35 798			
child age^b^	1432	3191	−4786	7319			
parent gender^c^	128 096	8691	111 310	143 909			
child gender^d^	−4939	9383	−23 889	12 125	−0.526	58.185	0.601
recording order^e^	−11 844	9185	−29 211	6302	−1.289	58.448	0.202
register:child age	10 011	3713	2926	17 373	2.696	59.798	0.009
register:parent gender	−24 256	8780	−41 326	−6979	−2.763	78.670	0.007

^a^ Dummy coded with ADS being the reference category.

^b^ *z*-transformed to a mean of 0 and an s.d. of 1, mean (s.d.) of the original variable were 356 (128.5) days.

^c^ Dummy coded with father being the reference category.

^d^ Dummy coded with boy being the reference category.

^e^ Dummy coded with ADS–IDS being the reference category.

### 3.6. Vowel space area (full version; model 6)

**Figure 9.**
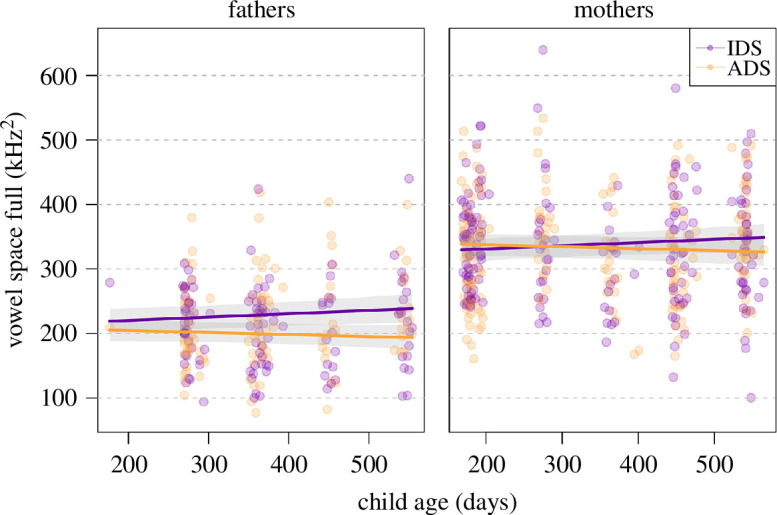
Vowel space area (full) as a function of register and child age. Coloured lines show the fitted final model (model 6) and the shaded areas its 95% confidence interval for all other predictors being at their average.

**Figure 10.**
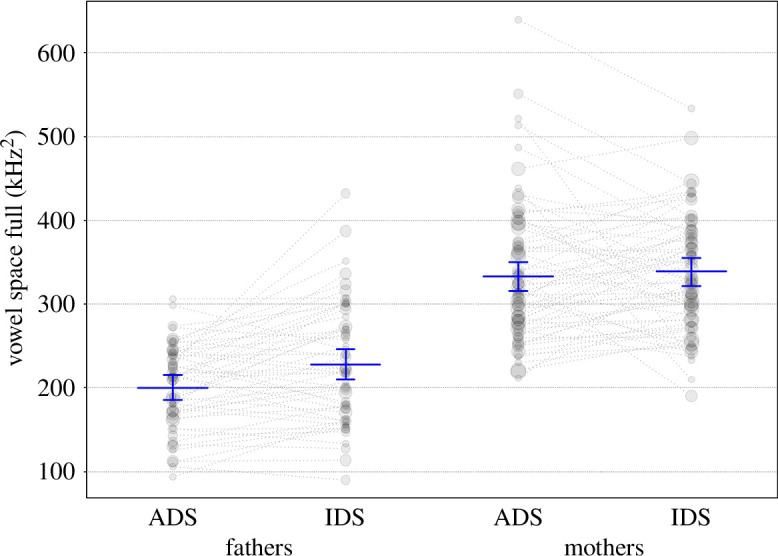
Vowel space area (full) as a function of register and parent gender. Horizontal blue lines show the fitted model (model 6) and vertical lines its 95% confidence interval for all other predictors being at their average, dotted lines connect individual parents and the area of data points reflects the number of observations, ranging from 1 to 5.

Overall, register (and its interactions, see below) had a clear impact on vowel space area as indicated by a likelihood ratio test comparing the full and null model (*χ*^2^ = 20.67, d.f. = 4, *p* < 0.001). After the removal of non-significant interactions (appendices 13a and 13b), we found that vowel space area (full) slightly increased in IDS with increasing child age, while it decreased in ADS (significant interaction; table 10; figure 9). Again, the figure depicts the interaction separately for mothers and fathers, although the three-way interaction with parent gender was not significant (a collapsed plot is provided in appendix 13c). Furthermore, fathers’, but not mothers’, vowel space area (full) was overall larger in IDS as compared with ADS (significant interaction; table 10; figure 10). The estimated results for the random effects of the full model are depicted in appendix 13d.

**Table 10.** Results of the reduced model (lacking the non-significant three-way and two-way interactions) with vowel space area (full version) in Hz^2^ as the response (model 6; estimates, together with s.e., confidence limits and significance tests).

term	estimate	s.e.	lower CI	upper CI	*t*	d.f.	*p*
intercept	212 171	11 526	189 165	235 115			
register^a^	28 498	8780	9823	44 740			
child age^b^	−3841	3199	−10 124	2671			
parent gender^c^	133 141	11 006	110 202	153 825			
child gender^d^	−3137	11 771	−26 116	21 157	−0.266	56.311	0.791
recording order^e^	−20 106	11 555	−44 236	841	−1.740	56.994	0.087
register:child age	10 149	3671	2818	17 098	2.765	82.114	0.007
register:parent gender	−22 517	10 993	−44 048	667	−2.048	73.891	0.044

^a^ Dummy coded with ADS being the reference category.

^b^ *z*-transformed to a mean of 0 and an s.d. of 1, mean (s.d.) of the original variable were 356 (128.5) days.

^c^ Dummy coded with father being the reference category.

^d^ Dummy coded with boy being the reference category.

^e^ Dummy coded with ADS–IDS being the reference category.

### 3.7. Vowel variability (model 7)

**Figure 11.**
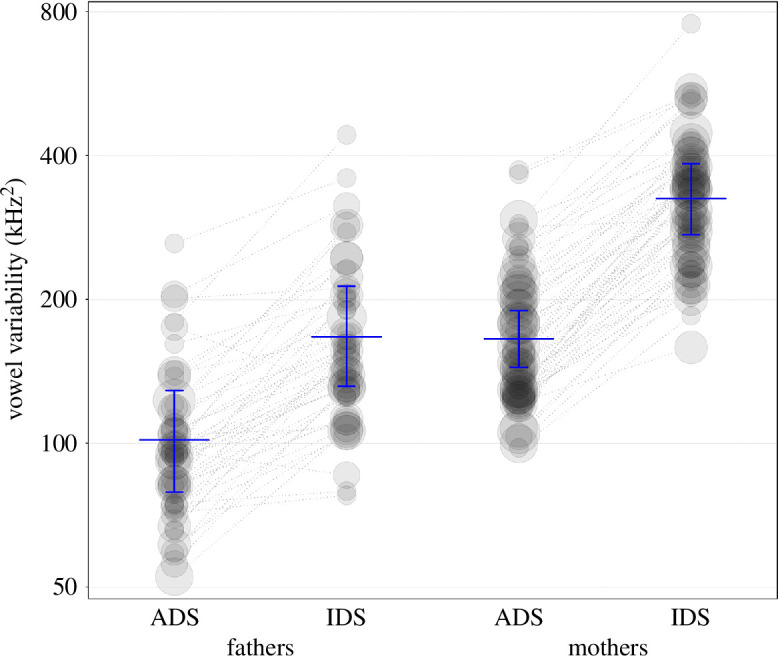
Vowel variability as a function of register and parent gender. Horizontal blue lines show the fitted model (model 7) and vertical lines its 95% confidence interval for all other predictors being at their average, dotted lines connect individual parents and the area of the data points reflects the number of observations, ranging from 9 to 45.

Overall, register (and its interactions, see below) had a clear impact on vowel variability, as indicated by a likelihood ratio test comparing the full and null model (*χ*^2^ = 27.40, d.f. = 4, *p* < 0.001). After the removal of all non-significant interactions (appendices 14b and 14c), we found that parents’ vowel variability increased in IDS as compared with ADS, and more so in mothers than in fathers (significant interaction; table 11; figure 11). The estimated results for the random effects in the full model are depicted in appendix 14d.

**Table 11.** Results of the reduced model (lacking the non-significant three-way and two-way interactions) with vowel variability (in log-transformed Hz^2^) as the response (model 7; estimates, together with s.e., confidence limits and significance tests).

term	estimate	s.e.	lower CI	upper CI	*t*	d.f.	*p*
intercept	11.498	0.124	11.227	11.746			
child age^a^	−0.010	0.014	−0.037	0.018	−0.721	42.771	0.475
register^b^	0.498	0.067	0.362	0.634			
parent gender^c^	0.487	0.093	0.287	0.674			
child gender^d^	0.032	0.057	−0.081	0.152	0.564	60.754	0.575
recording order^e^	0.022	0.059	−0.098	0.142	0.384	56.854	0.703
register:parent gender	0.178	0.064	0.038	0.308	2.799	30.468	0.009

^a^ *z*-transformed to a mean of 0 and an s.d. of 1, mean (s.d.) of the original variable were 358 (127.7) days.

^b^ Dummy coded with ADS being the reference category.

^c^ Dummy coded with father being the reference category.

^d^ Dummy coded with boy being the reference category.

^e^ Dummy coded with ADS–IDS being the reference category.

### 3.8. Vowel distinctiveness (model 8)

**Figure 12.**
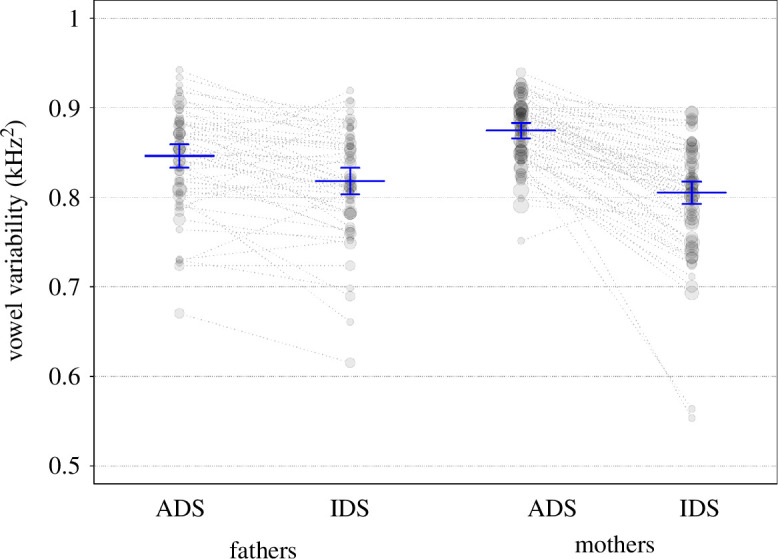
Vowel distinctiveness as a function of register and parent gender. Horizontal blue lines show the fitted model (model 8), vertical lines its 95% confidence interval for all other predictors being at their average, dotted lines connect individual parents and the area of the data points reflects the number of observations, ranging from 1 to 5.

Overall, register (and its interactions, see below) had a clear impact on vowel distinctiveness, as indicated by a likelihood ratio test comparing the full and null model (*χ*^2^ = 95.04, d.f. = 4, *p* < 0.001). After the removal of all non-significant interactions (appendices 15b and 15c), we found that parents’ vowel distinctiveness decreased in IDS as compared with ADS, and more so in mothers than in fathers (significant interaction; table 12; figure 12). The estimated results for the random effects of the full model are depicted in appendix 15d.

**Table 12.** Results of the reduced model (lacking the non-significant three-way and two-way interactions) with vowel distinctiveness (in quotients) as the response (model 8; estimates, together with s.e., confidence limits and significance tests).

term	estimate	s.e.	lower CI	upper CI	*χ* ^2^	d.f.	*p*
intercept	1.795	0.068	1.657	1.923			
child age^a^	0.017	0.017	−0.016	0.050	0.991	1	0.320
register^b^	−0.201	0.050	−0.293	−0.102			
parent gender^c^	0.237	0.062	0.114	0.361			
child gender^d^	−0.071	0.059	−0.191	0.054	1.432	1	0.231
recording order^e^	−0.089	0.057	−0.197	0.028	2.310	1	0.129
register:parent gender	−0.321	0.059	−0.443	−0.207	23.495	1	<0.001

^a^ *z*-transformed to a mean of 0 and an s.d. of 1, mean (s.d.) of the original variable were 356 (128.5) days.

^b^ Dummy coded with ADS being the reference category.

^c^ Dummy coded with father being the reference category.

^d^ Dummy coded with boy being the reference category.

^e^ Dummy coded with ADS–IDS being the reference category.

## 4. Discussion

The acoustic properties of IDS have been suggested to engage infants’ attention, foster socio-emotional bonding between infants and caregivers and facilitate infants’ language acquisition. However, the current literature is sparse as to how, and whether, parents modulate their IDS with infants’ age, potentially reflecting their child’s maturing social, cognitive and linguistic competencies. The aim of the current study was thus to investigate the intonational, temporal and segmental features of Norwegian parents’ IDS (in comparison to ADS), and the potential changes in these features across infancy, from infants who were 6–18 months old. We sought to extend on the current literature by adopting a relatively large sample size, measure a wide range of acoustic parameters, compare IDS and ADS that were sampled in a balanced design and tightly controlled for linguistic content and context between and within participants, using preregistered hypotheses and plans for analyses and a rigorous full-null model comparison approach to minimize type-I errors.

**Figure 13.**
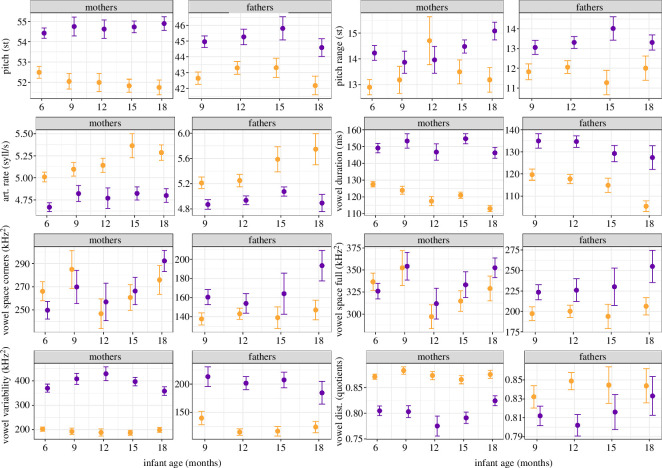
By-participant means for each acoustic measure by infant age (corresponding to T1–T5), grouped by register (IDS in purple, ADS in yellow) and parent gender. Note that dots represent the mean and vertical lines indicate the s.e. Only one father was part of the sample at 6 months and was thus omitted from the plot. Pitch and pitch range are in semitones, articulation rate in syllables per seconds phonation time, vowel duration in milliseconds, vowel space areas and vowel variability in kHz^2^, and vowel distinctiveness in quotients. The elevated average pitch range in mothers’ ADS at 12 months was caused by three participants with very low minimum pitch values.

Figure 13 provides a visualization of by-participant means for each acoustic measure by infant age, and table 13 provides a summary of our predictions for each acoustic measure, and whether they were supported by the study results. In brief, our hypotheses regarding the overall differences between registers were mostly confirmed, as Norwegian parents’ IDS differed systematically from their ADS on intonational, temporal and segmental features. However, the majority of our hypotheses regarding age-related trajectories of IDS were not supported, as several acoustic measures remained relatively stable across infants’ age, or had trajectories in the opposite direction of our predictions. We discuss the results of each acoustic measure in turn.

**Table 13.** Alignment of the study’s results with overall predicted register differences (IDS versus ADS) and age-related trajectories of IDS.

	predicted difference	supported	predicted trajectory of IDS	supported
pitch	higher in IDS	yes	decrease	no (stable)
pitch range	wider in IDS	yes	stable	no (increase)
articulation rate	slower in IDS	yes	increase	no (stable)
vowel duration	longer in IDS	yes	decrease	yes
vowel space area	increased in IDS	yes (fathers only)	stable	no (increase)
vowel variability	increased in IDS	yes	decrease	no (stable)
vowel distinctiveness	decreased in IDS	yes	increase	no (stable)

First, the intonational properties of Norwegian mothers’ and fathers’ IDS in our study shared the same characteristics as reported in most other languages studied to date [3], including previous studies of Norwegian parents [31,73]. That is, measured at the phrase level, IDS was spoken with a higher vocal pitch, and a wider pitch range, than ADS. These prosodic exaggerations were evident in both mothers and fathers, yet they might have been more subdued than what would have been found in spontaneously elicited speech [3]. Further, in IDS, pitch remained relatively stable across infants’ age, while pitch range increased. These results contrast with several longitudinal studies (of infants within our age range or with slightly older infants) that reported shifts towards ADS-like intonation in IDS with age [48,50,52,53], yet align with findings in, for example, Dutch-speaking mothers, whose pitch range was more exaggerated in speech to 15-month-olds than to 11-month-olds [17]. Potentially, exaggerated pitch height remains a tool to express positive affect, and draw and sustain infants’ attention, even later in infancy [14,41,54]. Producing speech with increasingly wide pitch range as infants develop could, in addition to its affective and attentional functions (cf. above), be a means for Norwegian parents to enhance speech contrasts (to more linguistically competent infants), given that Norwegian is a pitch accented language [123]. Although just a control variable, we also provide some interpretation for our finding that pitch (in ADS) and pitch range (in ADS and IDS) decreased as a function of later phrase onset within the speech recording. This could be due to parents adopting a wider pitch range as an initial strategy to attract the infant’s attention to the story and interactional setting, that fades by the end of the recording, or, given the similar pattern in ADS for both pitch measures, indicate that the varying content of the picture book (consisting of five individual stories) affected intonation patterns (e.g. the story of a sleeping cow at the end might elicit a soothing pitch pattern).

The temporal distinctions between IDS and ADS in our sample also adhered to prototypical characteristics of IDS, with slower articulation rate and elongated vowels [3,31]. More specifically, both mothers and fathers decreased the number of produced syllables per unit of phonation time during interaction with infants as compared with adults. However, articulation rate remained relatively stable in IDS across infants’ age. This is in contrast to previous studies that reported faster articulation rate in IDS with development [49,55,59,60], including studies with comparable infant age ranges and sampling frequencies as ours (e.g. six sessions from 4 to 14 months of age in [55]; four sessions from 7 to 24 months of age in [59]). There is some evidence that articulation rate in IDS during shared reading might be slower than in spontaneous speech [3]. Potentially, the book-reading interaction in our study might have been perceived by parents as a language learning opportunity for infants, as such maintaining their slow articulation rate even as infants became more linguistically competent, to ease cognitive demands [58,124,125]. Yet, parents slightly reduced their vowel duration in IDS with infants’ increasing age, a pattern not entirely consistent with the interaction being perceived as a didactic opportunity for word learning. The decrease in vowel duration with age, and the more exaggerated register-differences in vowel duration for mothers as compared with fathers, were, however, in line with our hypotheses, and with several previous studies of similarly aged infants [31,44,52,73], suggesting less emphasis on vowel duration as infants become more adept at processing speech over development. Moreover, vowels are the principal carriers of prosodic information, and caregivers might stretch their temporal dimension to clearly accentuate emotional valence in speech to younger infants, which is higher in positive affect [21,126], although whether this is related to differences between mothers and fathers is still unclear.

Finally, with respect to the segmental properties of vowels, our results echo previous reports of between-register differences, albeit with a disclaimer regarding vowel space expansion [3,67]. Namely, only fathers in our sample had larger vowel spaces in IDS, while mothers’ vowel spaces, overall, did not differ between IDS and ADS. Recall that we examined vowel space areas using two different measures: one based on the three corner vowels (in Norwegian: /i:/-/æ:/-/u:/) and one with all border vowels (/i:/-/e:/-/æ:/-/α:/-/ɔ:/-/u:/-/ʉ:/), as the latter would be more informative of infants’ overall vowel exposure. The absence of vowel space expansion in mothers’ IDS was apparent for both measures. Note that vowel space *reduction* had been reported in mothers’ IDS in several other languages, including Dutch to 11- to 15-month-olds [17], Cantonese to 3- to 12-month-olds [65] and Danish to 11- to 24-month-olds [68]. Yet, given that two recent studies from our laboratory demonstrated vowel space expansion in IDS to 8-month-olds [31] and to 18-month-olds [73], in both mothers and fathers, the lack of an effect in mothers here can, at first glance, appear puzzling. Yet, the analyses revealing that vowel space expansion in IDS, overall, increased with infants’ age suggest that both mothers and fathers contributed to this trend. Indeed, as can be seen in figure 13, while mothers in our sample demonstrated (numerical) vowel space reduction in IDS at 6 months, it shifts to vowel space expansion from 12 months on, an effect that appears to be quite robust at 18 months and in line with previous research on Norwegian parents [73]. Moreover, numerical reduction in vowel space observed in our mothers when infants were 6 months of age was also found in a previous study with Norwegian mothers to 0- to 6-month-old infants [127], providing supporting evidence for an absence of vowel space expansion in Norwegian mothers to very young 0- to 6-month-old infants. As such, the overall lack of an effect in mothers might be due to these dynamic changes with infant development and, potentially, to changes in the dynamics of parental involvement in childcare, as from 9 months on, in Norway, fathers^11^ are more likely to take care of the child with mothers having less caring responsibilities and interacting opportunities with the infant.

The linear increase in vowel space expansion with infants’ age contrasts with the majority of previous studies [3,45] (but see [64]) and suggests that Norwegian caregivers produce more peripheral vowel averages to infants as they are becoming increasingly more advanced language users, perhaps to facilitate their language learning [2,54,63]. However, our results also demonstrate that IDS, as compared with ADS, was characterized by more underlying variability in vowel production, and less distinctive/more overlapping vowel categories, that did not vary as a function of infants’ age. This is in line with previous studies of infants within the age range of our sample [68–70,72], including Norwegian [31], now collectively challenging the notion that IDS, in terms of phonemic realizations, is a clearer and didactic input signal, tailored to language learners (see also [28,129,130]). Further investigations are needed to discover the causes behind the seemingly more variable and less distinct internal distributions of vowels in IDS, which, interestingly, seem more apparent in mothers than in fathers. Potential candidates could be physiological and/or acoustic modulations caregivers do to increase positive affect and socio-emotional bonding, and which impact vowel formant frequencies [17,19,36,71]. Alternatively, analogously to prosodic exaggeration, variations in vowel production could be a means to engage infants’ attention through novelty.

Note that several limitations of our study must be acknowledged and considered in future research. First, while our aim was to compare the acoustic differences between IDS and ADS within a controlled linguistic context, we cannot rule out that the environmental conditions of the laboratory setting and the associated task demands of shared book reading may have influenced parents differently between registers. To date, within-participant studies examining this possibility remain limited, yet we remind readers that overall, effect sizes for some acoustic properties in the IDS literature differ as a function of setting and task [3]. Likewise, we can only speculate whether age-related trajectories of IDS would appear differently under less restricted elicitation methods. The content and structure of spontaneous caregiver speech might change with infant age (although the trajectories are not necessarily linear [131,132]), that could in turn impact acoustic characteristics. Next, increasing familiarity with the book stimuli over time could have had differential effects on IDS versus ADS, that might explain the age-related changes in pitch, articulation rate, vowel duration and vowel space area in ADS. Such familiarity effects could imply that the stability in IDS with infants’ age (yet changes in ADS) reflected adaptation to repeated exposure rather than inherent stability. However, while there is evidence that repeated reading of a text increases reading fluency (operationalized as ‘correct-words-per-minute’ [133]), it is unclear how this would manifest in our task and/or acoustic measures. Moreover, the majority of parents in our study participated in at most three sessions, with three months between consecutive sessions. Thus, we would not expect effects of repeated exposure to be very pronounced. Still, to circumvent familiarity effects—possibly also a confound in previous studies that used the same set of toys at each session—future work could introduce novel speech-elicitation stimuli at each session (although this would bring its own limitations with respect to between-session comparisons). In sum, the changes in acoustic parameters in ADS over time, which could also be caused by numerous other factors that impact speech (see §1), highlight the importance of systematically controlling for ADS in longitudinal studies of IDS through matched data collection of both registers. Further, in the current study, we analysed vowels in a limited set of 45 target words. While this number is greater compared with similar studies which often only use three targets, an extended pool of words would have increased the representativity of our vowel-based measures. Moreover, given that we recruited the ‘main caregiver at the time’, parents’ gender and infants’ age were confounded at several time points, in line with parental leave patterns, although our models accounted for this variability. While practically challenging, recording speech from both of the infants’ parents across time could further allow for an examination of gender effects on IDS, while controlling for age and parental leave status. Additionally, our sample was followed for one year, from 6 to 18 months of age. Clearly, investigating IDS over an even wider age range (consequently capturing a broader scope of infants’ development in various domains) might reveal different results. Finally, it is crucial to recognize that our sample primarily consisted of high SES families, with the majority of parents having master’s degrees, limiting the generalizability of our findings to other socio-economic—in addition to cultural and linguistic—contexts [134,135].

To conclude, in a longitudinal study of the acoustic properties of IDS and ADS, that controlled for linguistic context and used a relatively large sample size compared with previous studies, Norwegian mothers and fathers were found to make systematic acoustic modulations in their speech when interacting with their infant as compared with an adult. More precisely, parents adopted a higher pitch, wider pitch range, slower articulation rate and increased vowel duration. However, results for vowel space area were somewhat more complicated. Further, as compared with ADS, IDS was characterized by increased variability and decreased distinction of vowel categories. Together, this highlights that IDS is not across-the-board ‘clear’ input, and its expression might be primarily motivated by attentional and socio-emotional purposes. Moreover, speech directed to Norwegian infants is expressed with some acoustic features that are dynamic across development, and others that are static. Specifically, pitch, articulation rate, vowel variability and vowel distinctiveness remained relatively stable, while both pitch range and vowel space increased with infants’ age, and vowel duration slightly decreased. Future studies should investigate how the acoustic constants and variations influence the proposed attentional, socio-emotional, and linguistic functions of IDS.

## Data Availability

Data, code and materials are available at the Open Science Framework project page, also provided in the text [136]. Supplementary material is available online [137].

## Footnotes

^1^
And in several non-human species [7–9].

^2^
For example, infants could increase their attention to the speaking parent due to the positive affect of IDS, which, accidentally, might also lead to a more ‘learnable’ speech signal by vowel space expansion that arises from smiling (see discussions in [17,29]).

^3^
Bernstein [74] does not report infants’ age, but infants were at minimum 9 months of age at the pre-verbal stage, and the longitudinal design lasted for 6 months.

^4^
Note that the current study did not focus on dialectal differences, but on within-subject differences, over time, in acoustic features of speech addressed to the child as compared with an adult.

^5^
In Norway, parental leave constitutes either 49 weeks of leave with 100% salary, or 59 weeks of leave with 80% salary, out of which mothers and fathers are entitled to an equal amount [91].

^6^
Dialects are not used in written text; hence this is one of two official, dialect-neutral, written forms of Norwegian. However, our impression is that all parents naturally used their own dialect during reading.

^7^
This was considered comparable to an approach where we used individual formant ceilings per participant, thus fixed ceilings were used for consistency with previous studies on Norwegian IDS [31,73].

^8^
While not central to our hypotheses, this was to control for potential fatigue over the duration of a speech recording that could impact our acoustic measures.

^9^
Note that, for brevity, from here on out, we do not repeat references to statistical concepts and specific libraries and their functions and refer readers to their first mentions.

^10^
1700 ms was used to align the fitted model to our data (the average phonation time in the data was 1734.9 ms).

^11^
See [128] for an investigation of fathers’ IDS and parental leave experience.
